# Mechanical Homogenization Promoting Dual‐Directional Upcycling of Layered Oxide Cathodes

**DOI:** 10.1002/adma.202504380

**Published:** 2025-04-29

**Authors:** Nianji Zhang, Huan Li, Chao Ye, Shi‐Zhang Qiao

**Affiliations:** ^1^ School of Chemical Engineering the University of Adelaide Adelaide SA 5005 Australia

**Keywords:** battery recycling, bulk reconstruction, direct recycling, layered oxide cathode, upcycling

## Abstract

Upcycling is regarded as a sustainable and promising recycling solution for spent lithium‐ion batteries (LIBs). However, current upcycling strategies such as converting Ni‐lean to Ni‐rich cathodes struggle to change the composition of the spent cathodes to meet the diverse market demands. In addition, the commonly employed molten‐salts method requires tens of hours of high‐temperature treatment, restricting its sustainability. Herein, this study reports an efficient, flexible dual‐directional upcycling strategy to upcycle a broad family of layered oxide cathodes into fresh LiNi_x_Co_y_Mn_z_O_2_ (NCM) cathodes with tailored Ni‐contents—either increased or decreased—in just 4 h via mechanical homogenization pretreatment. This study confirms that the bulk diffusion of transition metals (TMs) is the rate‐determining step in the resynthesis process, and the mechanical homogenization can shorten the diffusion pathway of TMs, thus reducing the sintering duration effectively. The as‐upcycled NCM cathodes can deliver electrochemical performance on par with commercial counterparts. Notably, a systematic technoeconomic analysis shows that upcycling spent LiCoO_2_ into NCM622 can yield a profit up to 35 US$/kg, 30% higher than the conventional acid‐leaching resynthesis approach. This work provides an energy‐saving, widely adaptable, flexible, and cost‐efficient method for regenerating spent cathode materials, paving the way for the sustainable recycling of LIBs.

## Graphical abstract

This work introduces an efficient dual‐directional upcycling scheme enabled through a mechanical homogenization pretreatment. It enables various layered oxide cathodes to be reprocessed into fresh NCM cathodes with tailored Ni contents through boosted atomic diffusion in just 4 h of solid‐state sintering. Delivering upcycled cathodes with comparable electrochemical performances to their commercial counterparts, this approach excels itself from the cost‐effectiveness over conventional acid‐leaching resynthesis approaches.

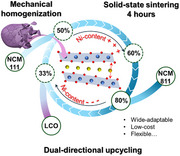

## 1 Introduction

Developing efficient recycling strategies for spent lithium‐ion batteries (LIBs) has become a critical factor to achieve carbon neutrality proposed by global community. Conventional recycling methods, including pyro‐ and hydrometallurgy, recover specific metals in spent LIBs. The primary advantage of these destructive techniques lies in their high tolerance for feedstock heterogeneity, allowing them to handle various spent cathode materials from different sources. Additionally, the extracted elements can be flexibly reconstituted into fresh cathodes with desired compositions, meeting the ever‐evolving market demands.^[^ ^1^ ^]^ For instance, spent LiCoO_2_ from portable electronics or LiNi_0.33_Co_0.33_Mn_0.33_O_2_ (NCM111) from e‐bikes (or even mixed with other compounds) can be reprocessed into desired NCM cathodes (e.g., Ni‐rich NCM622) to meet soaring demand for the electric vehicle sector. However, such adaptability and flexibility in conventional recycling methods is realized at the expense of weak sustainability caused by high energy and chemical inputs, hindering the achievement of closed‐loop recycling and carbon neutrality. Recently, non‐destructive recycling methods show great promise in maintaining sustainability, such as direct recycling^[^ ^2^ ^]^ for efficient restoration back to original performance and upcycling^[^ ^3^ ^]^ for greater flexibility and value.^[^ ^4^ ^]^ For instance, upcycling of spent LiCoO_2_ via doping foreign elements like Na,^[^ ^5^ ^]^ Ni/Mn,^[^ ^6^ ^]^ or Al/Mg^[^ ^7^ ^]^ has been shown to successfully elevate the working voltage of LiCoO_2_ to adapt the next‐generation high‐energy‐density LIBs.^[^ ^5^, ^7^, ^8^ ^]^

Beyond simple elemental doping, an alternative non‐destructive upcycling strategy—bulk reconstruction—can modify cathode chemistry.^[^ ^3a^ ^]^ It allows flexible production of cathodes with higher market maturity and demands.^[^ ^3^, ^8^ ^]^ Relevant reports proposed upcycling of Ni‐lean NCM cathodes into Ni‐rich NCM through molten‐salts method by feeding more Ni ions into the crystal lattice, which greatly enhances the energy density of upcycled cathode materials. However, the flexibility of this one‐direction upcycling strategy is still limited because the extra Ni injection based on solid‐phase diffusion causes the Ni content in the upcycled cathodes to rise with each recycling process. This greatly sacrifices their stability and safety, making them less suitable for wide applications. In technical aspect, the purpose of the molten salts is to provide a liquid flux with enhanced elemental diffusion^[^ ^8d^ ^]^; however, it remains inevitable to overcome the diffusion barriers inherent to the rigid lattice crystal and strong transition metal‐oxygen (TM‐O) bonds.^[^ ^9^ ^]^ Thus, this process typically takes tens of hours at an elevated temperature to allow the slow Ostwald ripening, diminishing its commercial viability due to the associated energy costs and environmental burden.^[^ ^8^, ^10^ ^]^ In addition, the molten‐salt method comes with challenges including the corrosive nature of the molten salts, the usage of excess Li‐salts, and additional processes such as water washing and post‐sintering (particularly for Ni‐rich cathodes).^[^ ^10^ ^]^ Therefore, it is still urgent to develop recycling methods that are widely adaptable and flexible with minimized energy and chemical input.

**Figure 1:**
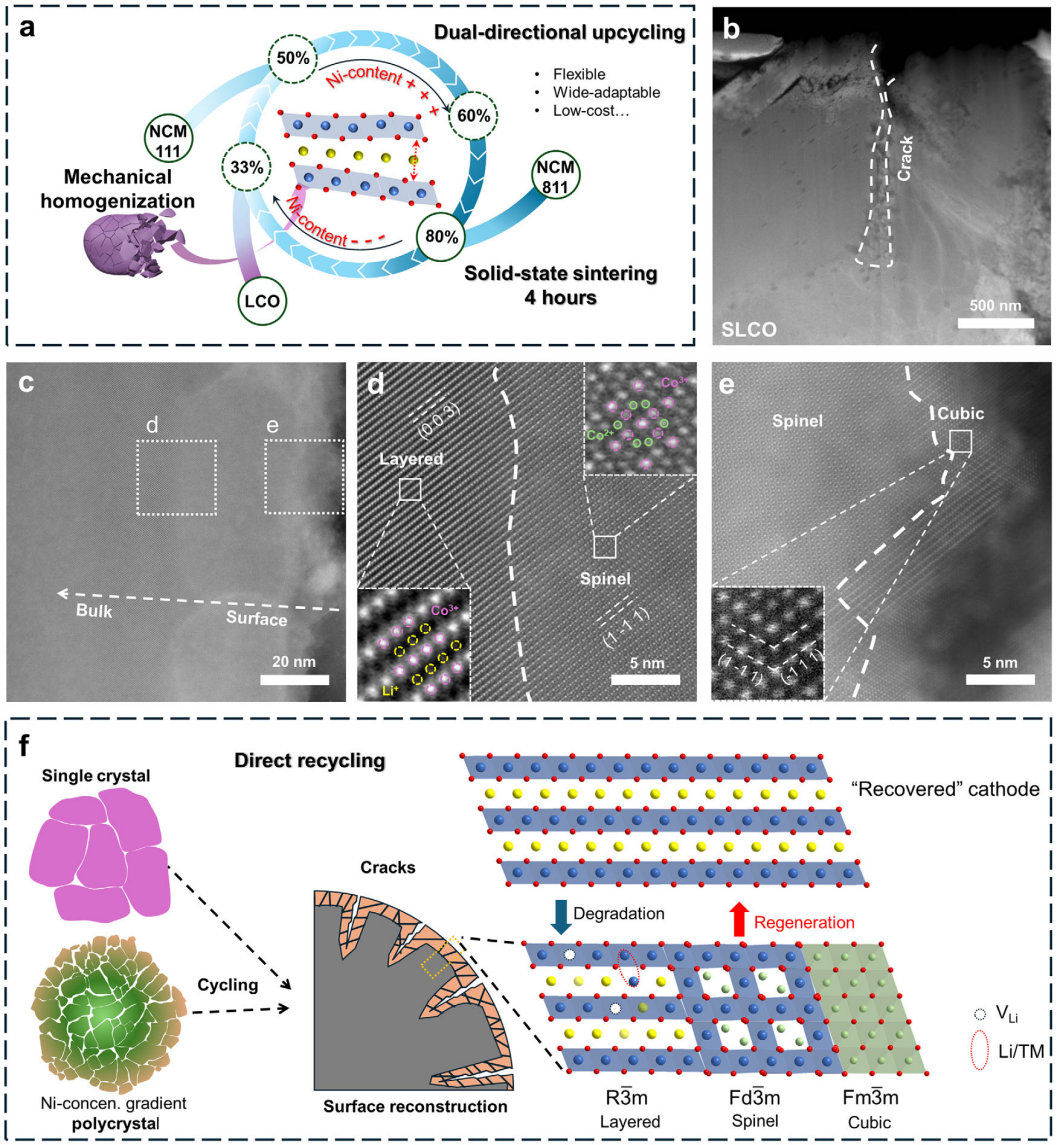
a) Schematic illustration of mechanical homogenization enabled dual‐directional upcycling process between varied Ni‐contents; b) Cross‐sectional scanning transmission microscopy (STEM) image of SLCO; c–e) Aberration‐corrected high‐angle annular dark field (HAADF)‐STEM images of SLCO at the area beside the crack; f) Schematic degradation mechanism of various cathode materials, particle to edge.

Herein, we report a dual‐directional upcycling strategy capable of upcycling various degraded layered oxide cathode materials, including LiCoO_2_, NCM111, and NCM811 through mechanical homogenization and subsequent sintering process. It can remanufacture fresh NCM cathodes with desired Ni contents—either increased or decreased from different precursors (i.e., dual‐directional upcycling)—in just 4‐hour solid‐state sintering (**Figure** **1**a). Ex situ X‐ray diffraction (XRD) studies confirm that the bulk diffusion of TMs is the rate‐determining step (RDS) in the bulk reconstruction of layered oxide cathodes, thereby rendering the liquid molten salts (promotes surface level diffusion only) largely ineffective for accelerating this process. The mechanical homogenization process can reduce the particle sizes of precursors, shorten diffusion pathways, and increase concentration gradients of added TM elements. The upcycled NCM cathodes exhibit the specific capacities of ≈156, 167, and 177 mAh g^−1^ for Ni‐content levels of 33%, 50%, and Ni‐rich 60%, respectively, comparable to those of their commercial counterparts. Moreover, benefited from simplified operation and resynthesis processes, this strategy is estimated to yield a profit up to 35 US$ kg^−1^ when the spent LiCoO_2_ upcycles into NCM622, 30% higher than the conventional acid‐leaching resynthesis approach. With wide adaptability to various layered oxide cathodes, this work enables a flexible, efficient, and economical closed‐loop upcycling to remanufacture fresh NCM cathodes with Ni‐content tailorable, opening new opportunities to meet evolving market demands.

## 2 Results and Discussion

### 2.1 Degradation Mechanisms and Dual‐Directional Upcycling of Layered Cathode Materials

The general degradation mechanism of the layered oxides cathode materials was first investigated using spent LiCoO_2_ (SLCO) as a representative to understand and develop the dual‐directional upcycling strategy. Both the scanning electron microscopy (SEM) and STEM imaging (Figure 1b; Figure S1, Supporting Information) clearly present the stress‐induced cracks in the SLCO particles. These cracks lead to the expose of extra cathode surfaces to electrolytes, resulting in serious side‐reactions and stimulating surface reconstruction.^[^ ^11^ ^]^ At the crack area (Figure 1c), HAADF‐STEM imaging demonstrate the surface reconstruction through a sharp contrast change from the surface to the bulk. As shown in Figure 1d,e and Figure S2 (Supporting Information), phases can be observed as cubic CoO at the surface, spinel Co_3_O_4_ in the subsurface, and layered Li_1‐x_CoO_2_ in the bulk. These are aligned with the results from the electron energy loss spectra (EELS) line scan shown in Figure S3 (Supporting Information), demonstrating the core‐loss Co *L_3_* peak shifting to higher energy and an increase of oxidation state.^[^ ^12^ ^]^ The presence of these degraded phases in typical spent cathodes underscores the significant structural and compositional deviations from the original layered structure (Figure 1f), resulting from the lattice collapse under lithium‐deficient status. Common direct recycling practices focus on relithiation of spent cathodes in a process akin to the conventional cathode synthesis which entails the reaction between metal oxides and lithium sources. However, it can only process cathode materials with singular chemistry, and the recovered cathode generally does not perform better than the original level.^[^ ^13^ ^]^

To realize the proposed dual‐directional upcycling strategy, mechanical homogenization (ball‐milling in this work) was employed to facilitate this process to upcycle various degraded layered oxide cathodes including SLCO, NCM111 (SNCM111) and NCM811 (SNCM811). As shown in Figure S4a–f (Supporting Information), all the spent cathodes were transformed into thin flakes with conspicuous size reduction after the ball milling process, allowing their homogeneous mixing with added Li/TMs salts in subsequent upcycling process. This effect results from the weaker interaction between the (003) planes in layered oxide cathode materials. The size reduction and lamellar morphology after ball‐milling are also evidenced by the broadened and intensity‐decreased (104) peaks on XRD patterns (Figure S4g–i, Supporting Information), respectively. Such size reduction with even cracks on these lamellar (Figure S5, Supporting Information) shortens the diffusion pathway for the externally added TMs, which steepens the concentration gradient, thereby promoting the diffusion effectively during sintering. Finally, six NCM cathodes with various Ni contents were successfully synthesized via mechanical homogenization as a pretreatment method and subsequent 4 h solid‐state sintering. These cathodes were designated as NCM111‐LCO‐BM, NCM111‐T811‐BM, NCM523‐LCO‐BM, NCM523‐T111‐BM, NCM622‐LCO‐BM, and NCM622‐T111‐BM, where “NCMxyz” indicates the final products of the upcycled cathodes, and “LCO”, “T111”, and “T811” specify the spent cathode materials from SLCO, SNCM111, and SNCM811, respectively. The suffix “BM” denotes ball milling as the processing method. For comparison, a control upcycling process was conducted using molten salts to produce NCM111 from pristine SLCO (NCM111‐LCO‐MS), where “MS” represents the molten‐salt method.

### 2.2 Physical and Chemical Properties of Upcycled Cathodes

Various characterization techniques were employed to determine the physical and chemical properties of upcycled cathodes, which play key roles in determining performance and value. Using the upcycling of SLCO into NCM111 as a case study, molten‐salts method was evaluated first as the control experiment. After sintering and water‐washing processes, the resultant NCM111‐LCO‐MS (Figure S6, Supporting Information) presented two sets of diffraction patterns of $R\bar{3}m$ space group that can be indexed as the NCM and LCO phases, respectively. Rietveld refinement was performed, and the results indicate 27.9 wt.% of LCO phase retained in NCM111‐LCO‐MS (Refinement details are included in Table S1, Supporting Information). Even extending the sintering duration to 10 h, it still maintained 19.6 wt.% of LCO (Table S2, Supporting Information). As shown in Figure S7 (Supporting Information), the SEM imaging demonstrates the NCM111‐LCO‐MS is made of highly irregular‐shaped particles forming aggregates with significant size variation, and energy‐dispersive X‐ray spectroscopy (EDX) mapping (Figure S8, Supporting Information) clearly evidences the presence of separate LCO phase while the NCM phase forms on the surface as small particles with a size of ≈100 nm. Considering the limited penetration depth of EDX, focused‐ion beam (FIB) was utilized to reveal the inner structure of NCM111‐LCO‐MS cathode, as shown in Figure S9 (Supporting Information). The cross‐sectional imaging reveals many inner voids, which are thought to have once contained molten salts before the water‐washing process. Furthermore, EELS mapping shows an inhomogeneous distribution of Co and Ni/Mn along the cross‐section, indicating the challenge to produce a homogeneous upcycled final product using the molten salts method.

**Figure 2:**
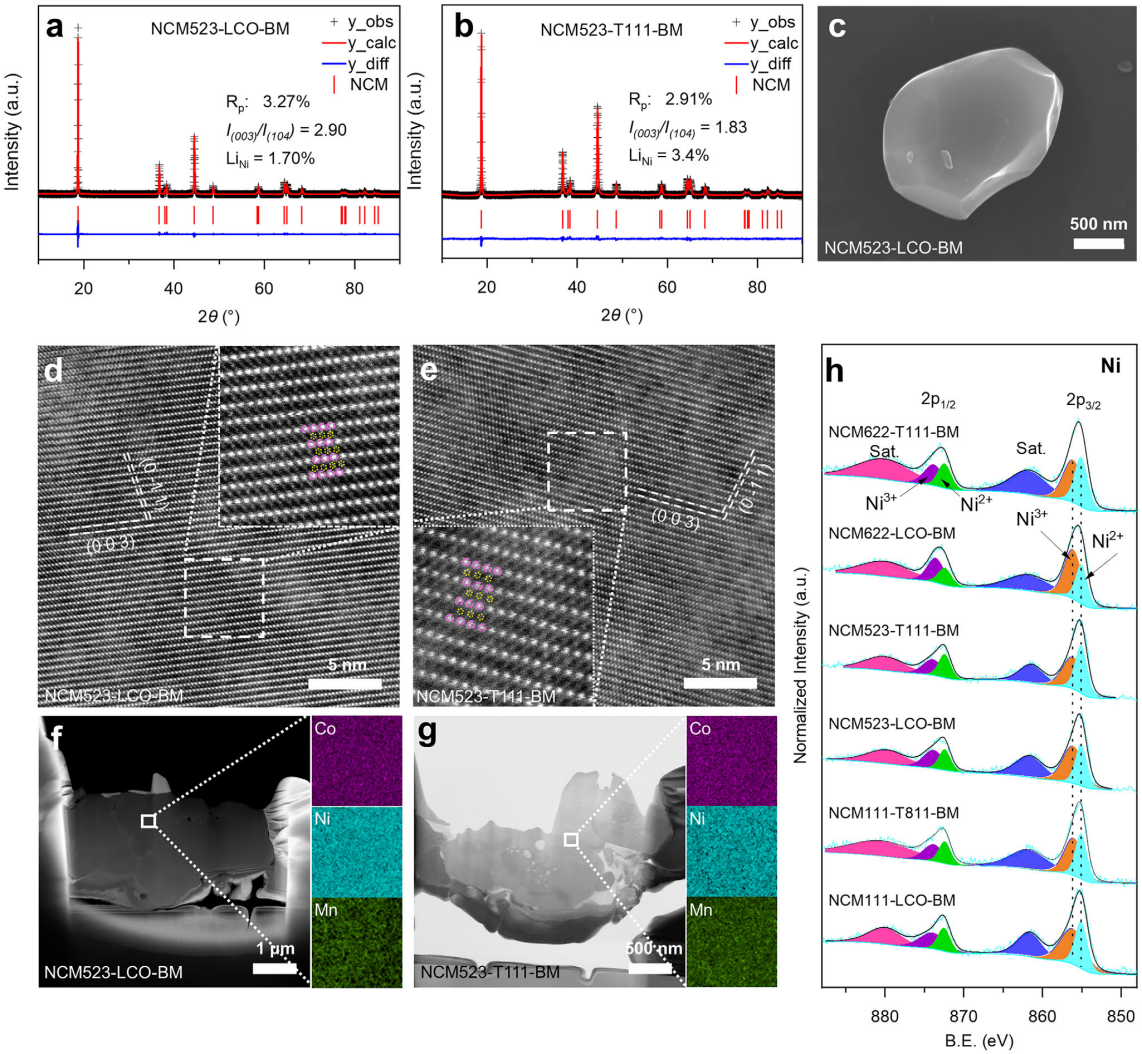
Rietveld refinement results of XRD diffraction patterns of a) NCM523‐LCO‐BM, and b) NCM523‐T111‐BM; c) SEM image of NCM523‐LCO‐BM; Aberration‐corrected HAADF‐STEM images of d) NCM523‐LCO‐BM and e) NCM523‐T111‐BM; STEM images and corresponding EELS mapping for Co, Mn, Ni elements in f) NCM523‐LCO‐BM, and g) NCM523‐T111‐BM; h) High‐resolution Ni 2p X‐ray photoelectron spectroscopy (XPS) spectra for upcycled NCM111‐LCO‐BM, NCM111‐T811‐BM, NCM523‐LCO‐BM, NCM523‐T111‐BM, NCM622‐LCO‐BM, and NCM622‐T111‐BM cathodes.

In contrast, XRD patterns of NCM cathodes upcycled via mechanical homogenization and 4‐hour solid‐state sintering (**Figure** **2**a,b; Figure S10, Supporting Information) show a single‐phase α‐NaFeO_2_ structure with well‐split (006)/(102) and (018)/(110) doublets, indicating good layered ordering. Meanwhile, Li/Ni disordering, caused by the similar radii of Li^+^ (0.76 Å) and Ni^2+^ (0.69 Å), is another critical measure to the NCM cathodes.^[^ ^3^, ^14^ ^]^ Such disordering will create larger electrostatic repulsion, and block the Li^+^ diffusion.^[^ ^15^ ^]^ Typically, Li/Ni disordering will greatly weaken the reflection from (003) plane, and it can be regarded as minor when the peak intensity ratio *I_(003)_/I_(104)_* is larger than 1.2.^[^ ^9^, ^16^ ^]^ All samples demonstrated *I_(003)_/I_(104)_* ratios ranging from 1.83 to 2.90, corresponding to the refined Li/Ni disordering degree from 3.38% to 0.84%, respectively. The reasonably small profile R‐factors (R_p_) of ≈3.0% and weighted profile R‐factors (R_wp_) of ≈4.5% from Rietveld refinement indicate a good agreement with the proposed O3‐type crystal structure (as shown in Tables S3–S8, Supporting Information). The morphology of these products was characterized using SEM (Figure 2c; Figures S11 and S12, Supporting Information). It can be observed that six NCM cathodes exhibit morphology of irregular shaped particles with particle sizes of ≈1 µm, few of which are aggregated. These are common phenomena attributed to the nature of solid‐state sintering. Figure 2d–g shows the HAADF‐STEM images of NCM523‐LCO‐BM and NCM523‐T111‐BM collected along [100] zone axis, both demonstrating highly ordered layered structure of the both samples. EELS mapping performed on STEM reveals homogeneous distribution of Ni, Co, and Mn in slices of NCM523‐LCO‐BM and NCM523‐T111‐BM prepared by FIB.

The surface electronic structure of the upcycled materials was further investigated using XPS. As shown in Figure S13a (Supporting Information), the full spectra of all six upcycled materials contain signals of Ni 2p, Co 2p, and Mn 2p orbitals, confirming the successful preparation of NCM cathodes. Moreover, the high‐resolution spectra of Ni 2p, Co 2p, and Mn 2p shown in Figure S13b (Supporting Information) were normalized to the Ni 2p peak intensity, with the Co 2p and Mn 2p peak intensities decreasing accordingly from Ni‐lean to Ni‐rich cathodes. The trend is also consistent with the varied composition of different upcycled materials. Deconvolved high‐resolution Ni 2p spectra of the upcycled materials are shown in Figure 2h. The peaks located at 855.16 and 873.07 eV are attributed to the Ni 2p_3/2_ and 2p_1/2_ orbitals, respectively. The deconvolved results are summarized in Table S9 (Supporting Information), and all the six upcycled cathodes display similar Ni^3+^/Ni^2+^ compositions with an average ratio value of 1.39, indicating the light Li/Ni disordering consistent with the results from XRD results. Apparently, these results demonstrate that the upcycled cathodes yielded by mechanical homogenization strategy show high phase purity, elemental homogeneity, and layered structural integrity, outperforming the molten‐salts technique. After upcycling, the elemental compositions of upcycled cathode materials (Table S10, Supporting Information) closely retain their intended Ni:Co:Mn stoichiometric ratios. The upcycled cathode materials (Figure S14, Supporting Information) exhibit high‐temperature thermal stabilities that are inversely proportional to Ni‐contents owing to the existence of high‐valence Ni^3+/4+^ ions.^[^ ^3a^ ^]^

### 2.3 Reaction Mechanism of Dual‐Directional Upcycling Process

**Figure 3:**
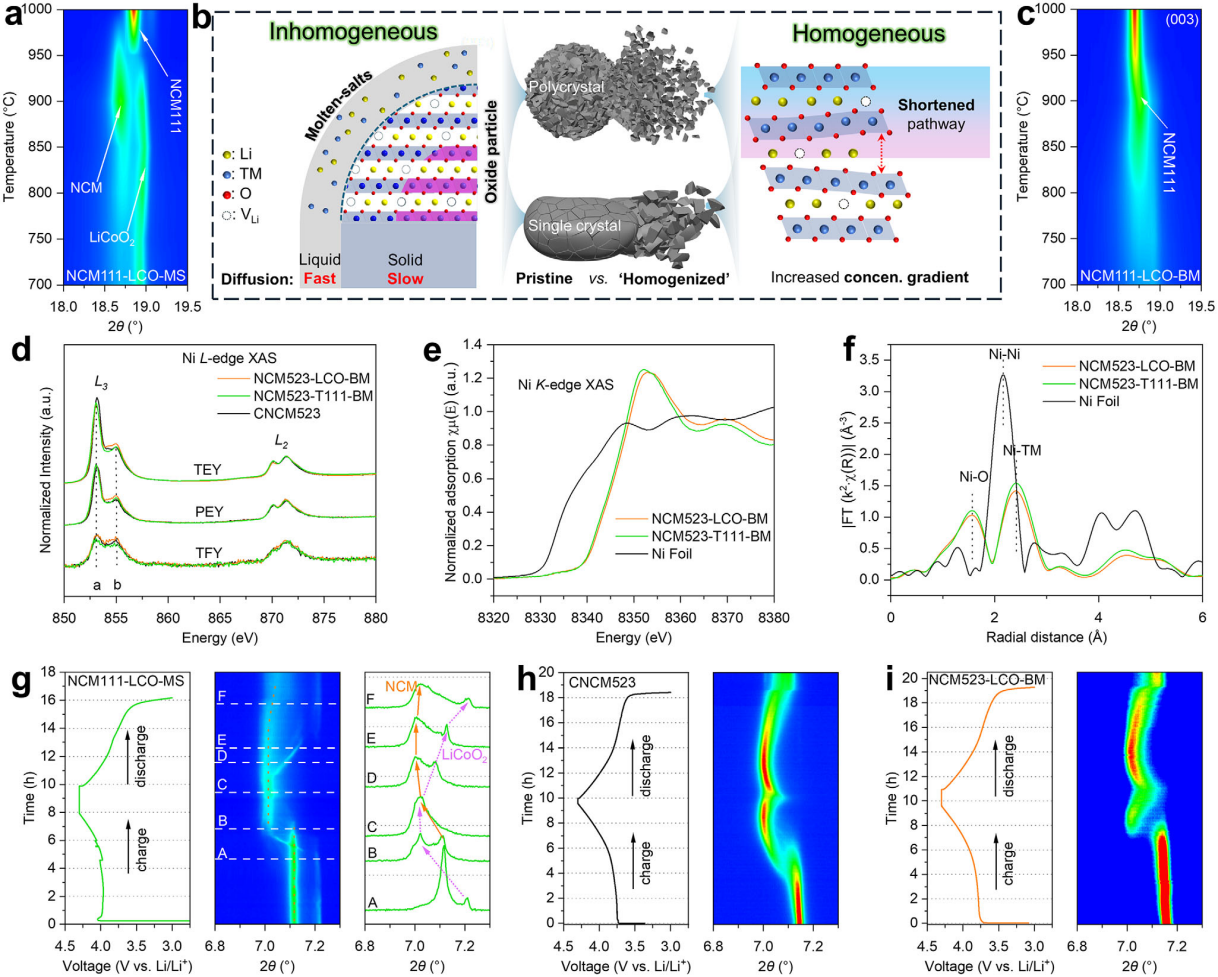
a) Enlarged lab‐XRD (003) peak evolution for NCM111‐LCO‐MS collected after sintering under 700–1000 °C, in an interval of 50 °C, for 4 h each; b) Schematic illustration for the elemental diffusion mechanism comparing molten salts method with the as‐proposed upcycling method; c) Ex situ XRD for NCM111‐LCO‐BM collected in same condition to a; d) Normalized soft X‐ray spectroscopy (SXR) spectra of the Ni *L_3_*‐edge of CNCM523, NCM523‐LCO ‐BM and NCM523‐T111‐BM in total electron yield (TEY), partial electron yield (PEY), and total fluorescence yield (TFY) modes; e) Normalized hard X‐ray absorption spectroscopy (XAS) of the Ni *K*‐edge for NCM523‐LCO‐BM and NCM523‐T111‐BM over the Ni ref. foil, and f) corresponding extended X‐ray absorption fine structure (EXAFS) R‐space curves; Operando synchrotron XRD measurements for g) NCM111‐LCO‐MS, h) CNCM523 and i) NCM523‐LCO‐BM cathodes under galvanostatic charging and discharging with a current density of 20 mA g^−1^ (0.1 C).

To comprehensively investigate the phase evolution and structural transformations during the sintering, ex situ XRD analyses were performed. A series of NCM11‐LCO‐MS samples were prepared by sintering for 4 h under various temperatures from 700 to 1000 °C, with an interval of 50 °C. As shown in **Figure** **3**a and Figure S15 (Supporting Information), the NCM111‐LCO‐MS system clearly exhibits a binary‐phase composition of NCM and LCO (from precursor SLCO) from 700 to 900 °C. The LCO phase remains largely unchanged up to 900 °C, while the intensity for NCM peaks gradually increases. Compared with TMs, the lower atomic mass of Li brings a higher diffusion rate, making it easier to react with TMs and form the layered structure. Only once the temperature rose to 950 °C did the peaks for LCO disappear, while new peaks (labeled as NCM111), lying between the NCM and LCO phases, emerged. However, the XRD result (Figure S16a, Supporting Information) indicates a co‐existence of NCM, NCM111, and LCO components at 950 °C. Given that the *c* lattice parameter of NCM lattice increases with Ni concentration,^[^ ^17^ ^]^ a conclusion can be drawn that the NCM phase is Ni‐rich but Co‐poor, while the Ni‐content of NCM111 phase is close to the target composition of 0.33. Under higher temperatures such as 1000 °C, the diffusion of TMs is greatly promoted yet the LCO phase can still be detected by XRD as shown in Figure S16b (Supporting Information). This can be explained by the fact that the benefit of molten salts lies in the boosted diffusion rates of TMs and Li ions within the liquid molten salts only (as schematically illustrated in Figure 3b). This explains the ineffectiveness of molten salts accelerating such upcycling process, in which the bulk diffusion of TM ions in the solid remains as the RDS. Consequently, NCM phases accumulated as fine particles on the surface, as observed in the SEM images of NCM111‐LCO‐MS (Figure S7, Supporting Information).

Accordingly, mechanical homogenization was introduced to facilitate this process by shortening the diffusion pathways of TM ions (Figure 3b). The increased concentration gradient enabled remanufacturing of NCM materials with varying Ni‐contents to be accomplished in shorter sintering duration, resulting in a more efficient upcycling process. As shown in Figure 3c, the NCM111‐LCO‐BM system exhibits a completely different response to high‐temperature treatment. With temperatures increasing from 700 to 1000 °C, it contains only one phase structure indexed as layered α‐NaFeO_2_‐type, i.e., NCM111. The crystal growth of NCM111 can be further evidenced by the splitting of the (018)/(110) peaks from 800 °C onward (as shown in Figure S17, Supporting Information), much lower than that (950 °C) of molten salts system begins splitting. These results demonstrate the benefit of mechanical homogenization in accelerating the dual‐directional upcycling process. Such phenomena can be explained from the shortened diffusion pathway of externally added TM elements from the particle surface to the core of the particle, enabling a boosted diffusion rate from increased concentration gradient.

Synchrotron‐based XAS techniques were utilized to investigate the transition metal valence state and electronic structure of the upcycled materials. The Ni *L*‐edge SXR spectra were collected in TEY, PEY and TFY modes, providing progressing detection depths from 5 to 100 nm.^[^ ^18^ ^]^ As shown in Figure 3d, the normalized Ni *L*‐edge SXR spectra of the upcycled NCM523‐LCO‐BM and NCM523‐T111‐BM both well overlap with the commercial NCM523 (denoted as CNCM523) across all detection modes, indicating the same distribution of Ni^2+^ in upcycled and commercial materials. The electronic structure and valence state of the bulk phase within the upcycled materials were further studied by hard XAS, as shown in Figure 3e,f. The NCM523‐LCO‐BM and NCM523‐T111‐BM demonstrate similar Ni oxidation states, meanwhile the EXAFS spectra of both samples display two similar peaks at 1.57 and 2.42 Å, corresponding to the path scattering of Ni‐O and Ni‐TM bonds, respectively.^[^ ^19^ ^]^ The above results indicate that the mechanical homogenization upcycled materials retained comparable electronic and structural characteristics to commercial material.

The structure evolution behaviors of the cathode materials make a determining role to the corresponding electrochemical performance in batteries. The operando synchrotron‐based XRD was carried out to investigate the evolution of crystal structures of NCM111‐LCO‐MS, CNCM523, and NCM523‐LCO‐BM during the 1^st^ charging and discharging processes. Figure 3g–i exhibits the voltage profile of three materials with a current density of 0.1 C (1 C = 200 mA g^−1^), with operando synchrotron‐XRD patterns of the characteristic (003) peak in 2D contour.

As aforementioned, the NCM111‐LCO‐MS contains two phases, the Co‐poor NCM and LCO, which are also revealed as double peaks at ≈7.12° and 7.21°, respectively, in the contour XRD patterns and stacked selected‐line plots (Figure 3g). When the NCM111‐LCO‐MS was charged to ≈3.97 V, a rapid (003) peak shift of the LCO phase from 7.20° to 7.0° was observed in the 2D contour (dash line A to B), while the NCM peak at 7.12° vanished slowly but it also shifted to ≈7.0° with further Li^+^ extraction (to dash line C). The combined peak at dash line C is asymmetrical with pronounced shoulder on the right side, which may come from the H2‐H3 phase transition from the LiCoO_2_ component. With discharging, the broad peak split into a main peak and a small peak: the first exhibited slight variation, shifting left before moving right, while the second returned to ≈7.2° suggestive of the LCO phase. This indicates that the NCM phase demonstrates poor reversibility, while LCO exhibits much better structure stability indicating the successful restoration in molten salts. The discharge capacity of NCM111‐LCO‐MS is only 125.77 mAh g^−1^ with a poor initial Columbic efficiency of only 76.46% (Figure S18a, Supporting Information). As shown in Figure S18b (Supporting Information), the differential capacity (*d*Q/*d*V) plot for NCM111‐LCO‐MS contains four pairs of redox peaks, the first pair (a/a′ at 3.84/3.67 V) comes from the NCM phase while the restored LCO phase contributes to the rest. It is easy to understand that the NCM phase in the inhomogeneous NCM111‐LCO‐MS contains a limited amount of Co, which contributes to the terrible lattice stability. These results suggest the importance of homogenous distribution of Ni, Co, and Mn elements in the upcycled NCM cathode. In contrast, NCM523‐LCO‐BM demonstrated similar structure evolution behaviors with commercial CNCM523. As shown in Figure 3h,i, upon charging to ≈4.0 V, the (003) reflection of both CNCM523 and NCM523‐LCO‐BM shifted to a lower angle. This shift resulted from lattice expansion along the *c*‐axis due to strengthened O─O repulsion as lithium content decreases.^[^ ^20^ ^]^ With further charging, the continued extraction of Li^+^ diminished the pillaring effect, causing the (003) peaks of both samples to shift to higher angles, indicating lattice contraction along the *c*‐axis. Meanwhile, the lattice parameter *a* for both samples kept decreasing with charging process owing to the shortened TM‐O distance and increased covalence from oxidation of TM ions (Figure S19, Supporting Information).^[^ ^21^ ^]^ During the first charge and discharge process, the maximum volumetric variation for NCM523‐LCO‐BM is 2.27%, closely matching the 2.10% observed for commercial CNCM523, highlighting the similar structural behavior and performance of mechanical homogenization upcycled cathodes with commercial ones.

### 2.4 Electrochemical Evaluations of Upcycled Materials

**Figure 4:**
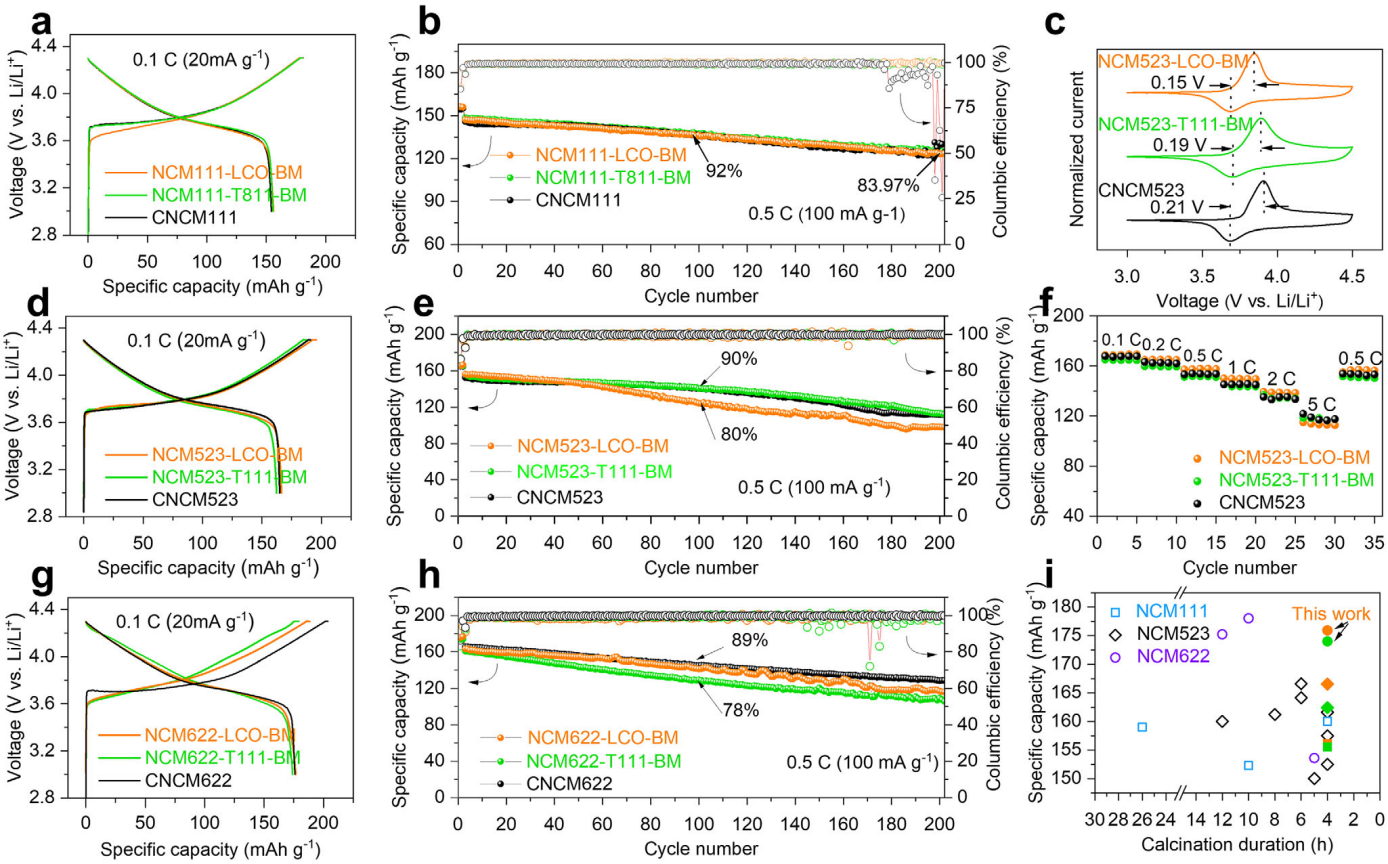
a) Charge and discharge curves and b) cycling stability of NCM111‐LCO‐BM, CM111‐T811‐BM versus CNCM111; c) CV curves, d) charge and discharge curves, e) cycling stability and f) rate capability for NCM523‐LCO‐BM, NCM523‐T111‐BM versus CNCM523; g) Charge and discharge curves and h) cycling stability of NCM622‐LCO‐BM, NCM622‐T111‐BM versus CNCM622 cathodes; i) Comparison of specific capacity over high‐temperature sintering duration required in this work and previous studies.^[^ ^8^, ^14^, ^22^ ^]^

The electrochemical performance of the upcycled materials plays a critical role in determining the viability of this strategy toward commercial applications. All upcycled materials were tested in a half‐cell battery configuration and benchmarked against commercial materials with equivalent nickel content under identical conditions. As shown in **Figure** **4**a, the upcycled NCM111‐LCO‐BM and NCM111‐T811‐BM demonstrated specific capacities of 156.16 and 155.54 mAh g^−1^, respectively, at 0.1 C (1 C = 200 mA g^−1^), while the commercial CNCM111 material presented a close discharge capacity of 154.27 mAh g^−1^. The upcycled NCM111 cathodes also displayed similar cycling stability to the commercial CNCM111 at 0.5 C, which all maintained over 82% of initial capacity after 200 cycles (Figure 4b). As shown in Figure S20 (Supporting Information), the upcycled materials also exhibited rate capabilities at the same level as CNCM111, even better at high rates. By contrast, the molten‐salts upcycled NCM111‐LCO‐MS delivered ≈120 mAh g^−1^ at 0.5 C which deteriorated quickly to only ≈60 mAh g^−1^ after ≈50 cycles (as shown in Figure S21a, Supporting Information), and the aforementioned “four redox peaks” property is also observed in *d*Q/*d*V and cyclic voltammetry (CV) plot (Figure S21b–d, Supporting Information). The galvanostatic intermittent titration (GITT) profiles and $D_{Li^{+}}$ evolutions of NCM111‐LCO‐MS are plotted in Figure S22 (Supporting Information), and different stages with disparate Li^+^ diffusion coefficients can be observed, indicating the separate contribution of LCO and NCM phases during the charging and discharging processes. Such results highlighted the great importance of creating homogeneous Ni/Co/Mn distribution within NCM cathodes.

This strategy can be also applied to prepare NCM cathodes with higher Ni‐content with competitiveness to commercial materials. As shown in Figure 4c–e, NCM523 materials upcycled from ball‐milling method delivered comparable electrochemical performances to commercial materials. The CV curves of NCM523‐LCO‐BM and NCM523‐T111‐BM showed similar redox peaks to those of CNCM523, but smaller voltage gaps between cathodic and anodic peaks indicating lower polarizations. As shown in Figure 4d, the upcycled materials also delivered specific capacities to 166.52 and 165.90 mAh g^−1^ at 0.1 C, respectively, exceeding 164.95 mAh g^−1^ of CNCM523. When cycled at 0.5 C (100 mA g^−1^), NCM523‐T111‐BM delivered similar cycling stability to CNCM523 (Figure 4e) in stark contrast to NCM523‐LCO‐BM experiencing accelerated degradation after only 50 cycles which may resulted from morphology of poorly aggregated and irregularly shaped particles. The upcycled materials also exhibited comparable rate capabilities to CNCM523, as shown in Figure 4f and Figure S23 (Supporting Information). The GITT profiles and $D_{Li^{+}}$ evolutions of NCM523‐LCO‐BM and NCM523‐T111‐BM are plotted in Figure S24 (Supporting Information), and NCM523‐LCO‐BM demonstrated higher $D_{Li^{+}}$ geomean value (1.09 × 10^−9^ cm^2^ s^−1^) than that of NCM523‐T111‐BM material (5.69 × 10^−10^ cm^2^ s^−1^), consistent to their Li^+^/Ni^2+^ disordering degree. Discharge specific capacities of 176.76 and 173.98 mAh g^−1^ were recorded for Ni‐rich NCM622‐LCO‐BM and NCM622‐T111‐BM (Figure 4g), respectively, comparable to the commercial benchmark CNCM622, which reached 175.89 mAh g^−1^ at 0.1 C. As shown in Figure 4h, NCM622‐LCO‐BM demonstrated similar cycling stability before the first 100 cycles with CNCM622, preserving ≈89% of initial capacity. Although NCM622‐T111‐BM showed a discharge‐specific capacity of ≈161 mAh g^−1^ at 0.5 C, slightly deteriorated compared to ≈163 mAh g^−1^ of CNCM622, it only retained 78% of initial capacity after 100 cycles. The rate performance of NCM622‐LCO‐BM was comparable to that of CNCM622 (Figure S25a,b, Supporting Information), yet NCM622‐T111‐BM presented poor rate capability at high charging rates, which may result from the undesirable Li/Ni^2+^ disordering as evidenced by XRD.

As summarized in Figure 4i, compared to the relevant literature on direct recycling, our proposed strategy produced three types of NCM cathodes, ranging from Ni‐lean to Ni‐rich, using three different types of degraded oxide cathodes, respectively, with performances greatly improved from the degraded oxide cathodes (Figure S25c, Supporting Information). This approach yielded materials with electrochemical properties that are amongst the best in the reported literature. To further investigate the potential for practical applications, NCM111‐LCO‐BM||Gr pouch cells (Figure S26, Supporting Information) were assembled. Cycling in the voltage range of 2.5–4.2 V at 0.5 C, the upcycled NCM111‐LCO‐BM material (Table S11, Supporting Information) is estimated to offer a gravimetric energy density of ≈185 Wh kg^−1^, reaching the commercial level. Moreover, feasibility validation of this strategy has also been conducted on air‐synthesized LiNiO_2_ (LNO‐Air), which presented (Figure S27a, Supporting Information) relatively higher Li^+^/Ni^2+^ disordering degree (*I*_*(003)*_/*I* *_(104)_* = 1.34) owing to the lower oxidation ability of air to oxidize Ni^2+^. After the mechanical homogenization enabled upcycling (Figure S27b,c, Supporting Information), Rietveld refinement indicates that the as‐obtained NCM111‐LNO‐BM has a Li^+^/Ni^2+^ disordering degree to only 1.7% (Table S12, Supporting Information), which confirms the wide‐adaptability of this strategy on layered oxide cathodes.

### 2.5 Technoeconomic Analysis

**Figure 5:**
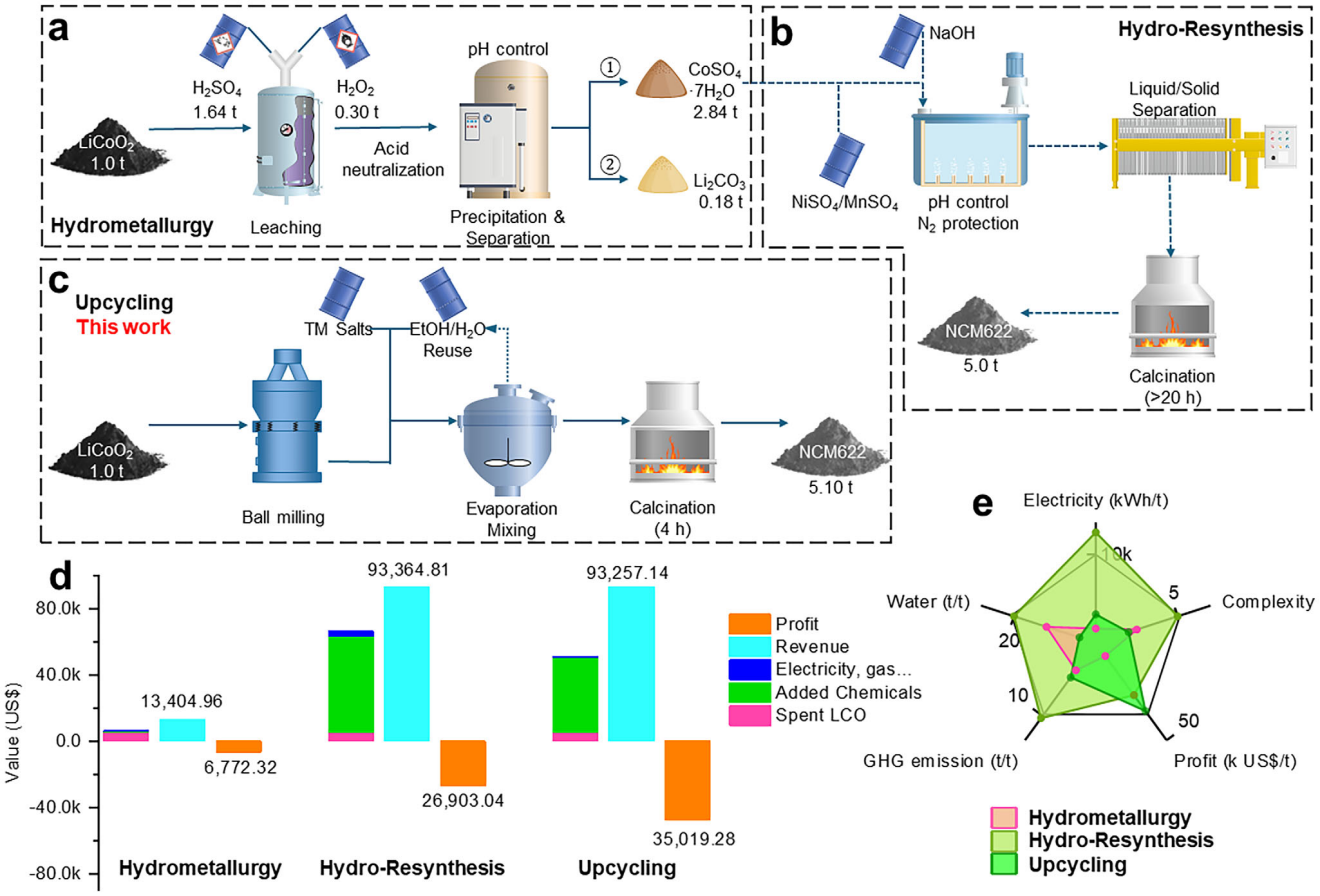
Material flow schemes of the a) Hydrometallurgy, b) Hydro‐Resynthesis, and c) Upcycling (this work) processes; d) Economic breakdown and analysis of Hydrometallurgy, Hydro‐Resynthesis and Upcycling processes; e) Comparison of the three different recycling schemes.

To evaluate economic viabilities of the dual‐directional upcycling process, we performed a systematic technoeconomic analysis comparing the proposed strategy with traditional hydrometallurgy and the essential resynthesis (Hydro‐Resynthesis to re‐manufacture cathode from the extracted salts), as shown in **Figure** **5**a–c.

In the traditional hydrometallurgical recycling process, sulfuric acid (H_2_SO_4_) and hydrogen peroxide (H_2_O_2_) are typically used to process spent cathode materials (Figure 5a). As detailed in Table S13 (Supporting Information), recycling 1 ton of spent lithium cobalt oxide (Li_0.6_CoO_2_, valued at ≈$5278.57 per ton) requires ≈1.64 tons of sulfuric acid and 0.30 tons of reducing agent H_2_O_2_, as calculated based on Equation S1 (Supporting Information). Moreover, the leaching process consumes ≈775 kWh of electricity to heat the solution to 80 °C, thereby optimizing the leaching rate. The resulting extraction produces 2.84 tons of CoSO_4_·7H_2_O and Li_2_CO_3_, which yield revenue of US$11468.72 and US$1936.24, respectively. This process also produces a lot of wastewater enriched with salt and acids, resulting in extra treatment costs. However, for resynthesis, the CoSO_4_ solution can be directly utilized in co‐precipitation process to produce TM(OH)_2_ precursors (Figure 5b). This process utilizes costly salts, such as NiSO_4_ and MnSO_4_, which add significant cost but also introduce greater technical challenges, particularly in maintaining precise pH control and optimizing reactor conditions. To produce Ni‐rich NCM622 cathodes, expensive lithium hydroxide (LiOH) is required in the step of high‐temperature lithiation, as the recycled Li_2_CO_3_ cannot be used. The synthesis involves a prolonged sintering process (up to 20 h) in an oxygen flow, consuming more than 12000 kWh of electricity. Ultimately, this approach produces ≈5.00 tons of NCM622, generating revenue of up to US$93364.81. Although the substantial chemical and energy costs make the Hydro‐Resynthesis process much more expensive than basic hydrometallurgy, it adds significant value, increasing the profit from US$6772.32 to US$26903.04 after resynthesis (Figure 5d).

In contrast, our proposed upcycling process offers a more cost‐effective and energy‐efficient solution by bypassing many of the chemical‐intensive steps found in the traditional methods. Through a mechanical homogenization method, particle size of the spent LCO can be greatly reduced with electricity consumption ≈160 kWh; moreover, the high‐temperature treatment can be shortened to only 4 h, further reducing energy consumption. Compared to Hydro‐Resynthesis, the upcycling process reduces chemical input costs to US$45511.64, which is 21.53% cheaper. As a result, the proposed upcycling method in this work generates a profit to 35 US$/kg, increasing 30.17% compared to the Hydro‐Resynthesis approach (Figure 5d). Moreover, upcycling's shorter treatment processes would lower the overall expenses that are not estimated here, including those related to equipment, labor, and other fixed costs. In addition to its economic benefits, the upcycling process developed in this work also demonstrates a lower environmental impact without the tedious co‐ precipitation and energy intensive resynthesis processes (Figure 5e; Tables S14–S16, Supporting Information). Benefiting from the shortened high‐temperature treatment, the as‐demonstrated upcycling approach delivers with correspondingly 4 times lower greenhouse gas emissions, reducing electricity and water consumption. Therefore, the proposed dual‐directional upcycling strategy with mechanical homogenization enabled a closed‐loop upcycling of various layered oxide cathodes, along with simplified operation processes, enhanced economic potential, and reduced energy consumption, evidencing its potential for practical applications.

## 3 Conclusion

In summary, this work demonstrated a dual‐directional upcycling strategy for a wide range of layered oxide cathodes (from LiCoO_2_ to NCM811) through simple solid‐state sintering. By overcoming the rate‐determining bulk diffusion of transition metals, the mechanical homogenization pretreatment allowed a rapid (4 h) remanufacturing of NCM cathodes with tailorable Ni contents (33–60 wt.%), while preserving a homogenous element distribution and electrochemical performance rivaling commercial materials. Besides, the homogenization process standardizes the spent cathodes by erasing morphological differences and weakening the composition variation, enabling the regeneration of commercial blended cathodes and enabling improved adaptability and flexibility in this work. Notably, this strategy was estimated to offer a commercial profit up to 35 US$ kg^−1^ to upcycle spent LiCoO_2_ into fresh NCM622 material, ≈30% higher than that obtained from conventional hydrometallurgy with cathode resynthesis. Our work paves the way for flexible, cost‐effective, and efficient dual‐directional upcycling of degraded layered cathodes from spent LIBs, addressing the evolving market demand for NCM cathodes with varied range of Ni contents.

## Conflict of Interest

The authors declare no conflict of interest.

## Supporting information



Supporting information

## Data Availability

The data that support the findings of this study are available from the corresponding author upon reasonable request.
