# Supplementary material for: Mechanical Homogenization Promoting Dual‐Directional Upcycling of Layered Oxide Cathodes
Source: Adv Mater. 2025 Apr 29;37(29):2504380. doi: 10.1002/adma.202504380 (PMC12288807; doi:10.1002/adma.202504380)
Supplement: Supplementary file 1 — Supporting information [file ADMA-37-2504380-s001.docx]

**Supporting Information**

for

Mechanical Homogenization Promoting Dual-Directional Upcycling of Layered Oxide Cathodes

*Nianji Zhang, Huan Li, Chao Ye,^*^ Shi-Zhang Qiao^*^*

N. Zhang, H. Li, C. Ye, and Prof. S.-Z. Qiao

School of Chemical Engineering, the University of Adelaide, Adelaide, SA 5005, Australia Email: chao.ye@adelaide.edu.au; s.qiao@adelaide.edu.au

# Experimental Section

**Chemical and materials**

All reagents were obtained from commercial sources without any purification. Lithium hydroxide (99%), Li_2_CO_3_ (99%), nickel hydroxide (99%), manganese acetate tetrahydrate (99%) and nickel acetate tetrahydrate (98%+) are all in analytical reagent grade and purchased from Sigma-Aldrich. Commercial NCM111, NCM523, NCM622, and NCM811 are purchased from Canrd.

**Collection of degraded cathodes**

Spent LCO cathode material is collected from cylindrical 18650 Li-ion batteries (Sanyo UR18650FM, 2.5 Ah) which are cycled between 3.0–4.2 V (CC-CV mode) with a charge/discharge current of 1.25 A (0.5 C) for 500 cycles. After soaking in K_2_SO_4_ solution overnight, the discharged 18650 battery is manually disassembled, and the cathode sheet is harvested. NMP is used to remove the black mass from the aluminum foil, and the spent LiCoO_2_ powder (SLCO) is harvested after sintering in air at 600 ℃ for 2 hours to remove PVDF and conductive carbon.

Degraded NCM111 was harvested from chemical delithiated CNCM111 after reaction with 25 mol.% K_2_S_2_O_8_ in water. After stirring overnight, the powder (SNCM111) is collected through centrifugation and dried in vacuum under 80 ℃. SNCM811 is obtained after exposing CNCM811 in air over a month.

**Mechanical homogenization enabled upcycling process**

The degraded CAMs are ball-milled for 20 hours under 300 rpm in anhydrous ethanol using ZrO_2_ milling media (3/5/10 mm) in a 50 mL Al_2_O_3_ vial. After milling, the powders are collected and dry at 80 °C, resulting in samples denoted as SBC (SLCO), SBT (SNCM111), and SB8T (SNCM811), respectively. The electrochemical performance of these precursors is illustrated in **Figure S18**d.

Firstly, stoichiometric amounts of Ni(Ac)_2_·4H_2_O and Mn(Ac)_2_·4H_2_O are dissolved in a 1:1 mixture of deionized water and ethanol. The ball-milled degraded CAMs powder is then added to the solution with continuous stirring until well-mixed. The resulting suspension is subjected to rotary evaporator to remove all solvents. The obtained powder is subsequently sintered under 500 ℃ for 1 hour in air to pre-oxidize. Then, a stoichiometric amount of lithium salt is manually ground with the pre-oxidized powders. Finaly, the mixture is calcinated under high temperatures for 4 hours to finish the upcycling process. NCM111 and NCM523 cathodes are prepared under 900 ℃ in air with Li_2_CO_3_, while NCM622 is prepared under 850 ℃ in oxygen with LiOH. An extra 5% molar of Li is used to compensate the Li-loss in high-temperature treatment.

**Molten-salts upcycled NCM111-LCO-MS**

The SLCO are mixed with stoichiometric amounts of Ni(Ac)_2_·4H_2_O and Mn(Ac)_2_·4H_2_O using the aforementioned method. After pre-oxidation under 500 ℃ for 1 hour in air, the obtained powders are mixed with stoichiometric amount of LiOH as well as excess 30 mol.% LiOH and 20 mol.% Li_2_SO_4_. Then, the mixture is subjected to high-temperature heat treatment for 4 hours under temperatures ranging from 700 to 1000 ℃. Besides, longer heat treatment under 900 ℃ for 10 hours is also performed. After temperature cools down naturally, the obtained powder is grinded manually and sent for water washing to remove the excess Li salts. Finally, the cathode is dried in vacuum under 60 ℃ overnight, and is denoted as NCM111-LCO-MS.

**Synthesis of LNO-Air and NCM111-LNO-BM**

Ni(OH)_2_ purchased from Sigma-Aldrich was manually mixed with 1.05 mol. times of LiOH·H_2_O in agate mortar, and then the mixed powder was directly sent for calcination under 850 ℃ for 8 hours in air. For synthesis of NCM111-LNO-BM, all other procedures are exactly same as aforementioned but adds Co(Ac)_2_·4H_2_O and Mn(Ac)_2_·4H_2_O.

**Electrochemical measurements**

The cathode active materials are well mixed with SuperP and PVDF (5 wt.% dispersed in NMP) in mass ratio of 85:7.5:7.5 to make the slurry. The slurry is then blade-coated onto carbon-coated aluminum foil and vacuum-dried at 80 °C for 10 hours. After drying, the foil is punched into circular discs (Ø 12 mm) with an active material loading of 5.00–7.00 mg/cm^2^. The electrochemical performance of the upcycled materials is evaluated using CR2032-type coin cells assembled in an Ar-filled glovebox, maintaining O_2_ and H_2_O levels below 0.1 ppm. Celgard 2500 film (Ø 16 mm) is used as the separator. Each cell contains 70 µL of electrolyte consisting of 1 M LiPF_6_ in a 1:1:1 vol. ratio of EC/DEC/DMC. Electrochemical testing of the coin cells is performed using Neware multichannel battery testers at 28 °C, with voltage ranging between 3.0 and 4.3 V. The charging cycle operates in a constant current-constant voltage (CC-CV) mode, while discharging is conducted at constant current (CC). Cyclic voltammetry (CV) is performed at a scan rate of 0.1 mV/s over a voltage range of 3.0–4.5 V using a Biologic potentiostat. The galvanostatic intermittent titration technique (GITT) was employed, incorporating a ten-minute current pulse at a rate of 0.2 C, succeeded by a rest period of one hour. Pouch cells (NCM111-LCO-BM||Gr) are assembled in an Ar-filled glovebox, maintaining O_2_ and H_2_O levels below 0.01 ppm. The cathode exhibits a loading of ~ 12 mg/cm^2^ with a tap density of 2.5 mg/cm^3^, while the anode has loading of ~5.7 mg/cm^2^ and a tap density of ~1.5 mg/cm^3^. Graphite and NCM111-LCO-BM electrodes with a size of 7 × 8 cm were used for the assembly of laminated pouch cells. The galvanostatic charge/discharge measurements of pouch cells were recorded the Land battery tester in a voltage range between 2.5-4.2 V.

**Characterization techniques**

The crystal structure of the samples is tested on an Advance D8 (Brucker) X-ray diffractometer equipped with a Cu Kα X-ray source (λ = 1.5418 Å). XRD data refinement is conducted on GSAS-II.^[1]^ The morphology of the cathode materials is observed on a field-emission scanning electron microscope (FE-SEM, HITACHI SU7000) equipped with an energy dispersive X-ray detector (EDX, Oxford). X-ray photoelectron spectroscopy (XPS) measurements are carried out on Escalab Xi+ (Thermo Scientific) with Al Kα radiation as X-ray source. Focused ion beam (FIB) milling is conducted on FEI DualBeam FIB-SEM with a Ga^+^ ion beam. Scanning transmission electron microscopy (STEM) imaging is performed on FEI Titan Themis with aberration correction on probe forming lens under 200 kV, equipped with a EDX detector and a Gatan Quantum GIF 965 electron energy loss (EELS) spectrometer. Composition of upcycled cathodes is determined by Atomic Absorption spectrometers (AAS, Shimadzu AA-6300), using Air:C_2_H_2_ flame with proper digestion of cathode powders in HCl solution and dilution to ppm level. Thermogravimetric Analysis (TGA) and Differential Scanning Calorimetry (DSC) curves are recorded on a STARe System TGA/DSC 3+ (Mettler Toledo) in a temperature range of 40 to 900 ℃ in N_2_ (30 mL/min) and temperature increasing rate of 5 ℃/min. In-situ electrochemical charge and discharge tests are performed in CR3032 coin cells with casings punched a 6 mm window and sealed by Kapton film, and data is collected from synchrotron Powder Diffraction beamline of Australian Synchrotron, part of ANSTO. The synchrotron XRD is collected under 21 keV with a wavelength of 0.5903 Å. Elemental *L*-edge and *K*-edge data are collected from soft X-ray spectroscopy (SXR) and X-ray adsorption spectroscopy (XAS) beamtime of Australian Synchrotron, part of ANSTO.

# Supplemental Figures

**
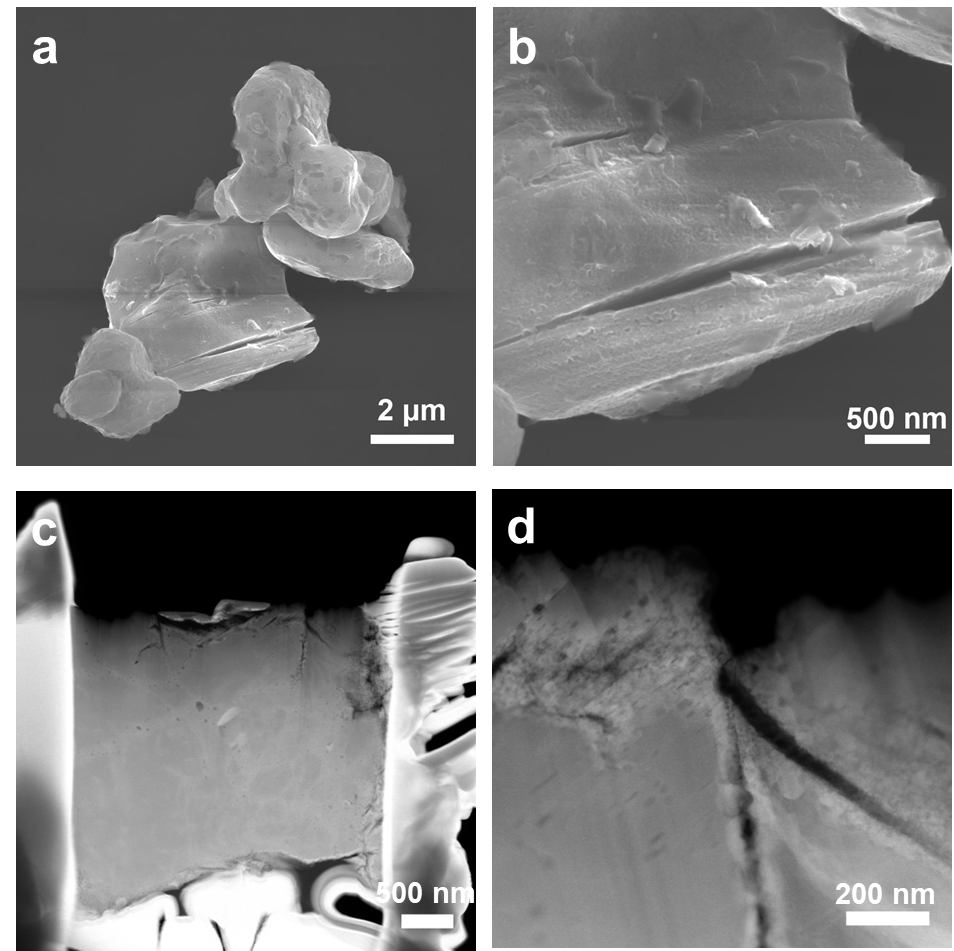
**

**Figure S1. a, b)** SEM images for SLCO; **c, d)** Cross-sectional STEM images for SLCO.

**
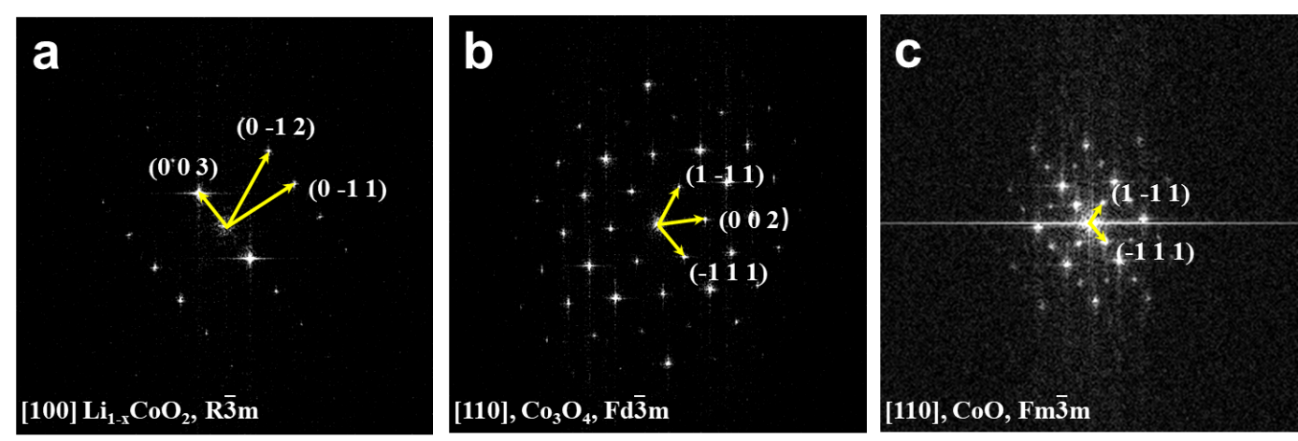
**

**Figure S2.** Fast Fourier Transformation (FFT) results for **a)** bulk layered $R\bar{3}m$(LiCoO_2_) structure, **b)** sub-surface spinel $Fd\bar{3}m$(Co_3_O_4_) structure, and **c)** surface rock-salt $Fm\bar{3}m$ (CoO) structure.


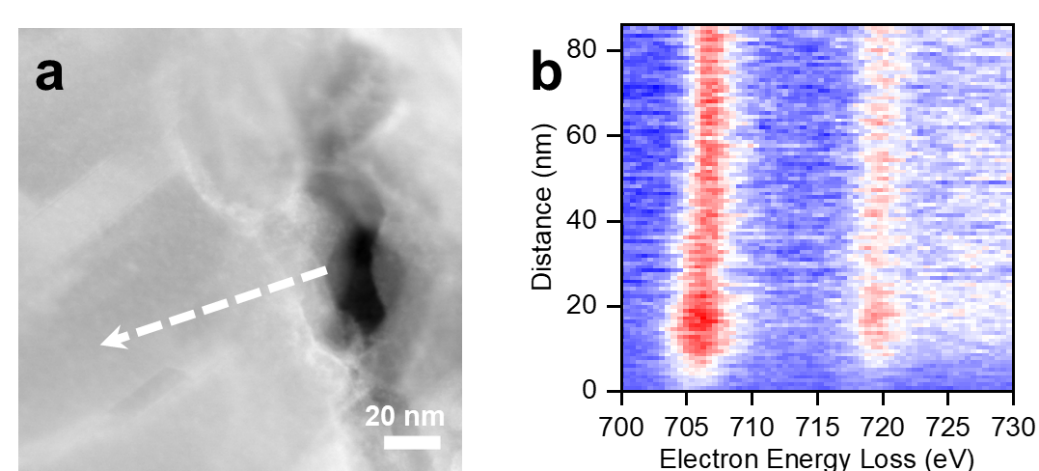


**Figure S3 a, b)** EELS line scan illustrating the Co *L_2,3_* peak shifts from surface to bulk.

**
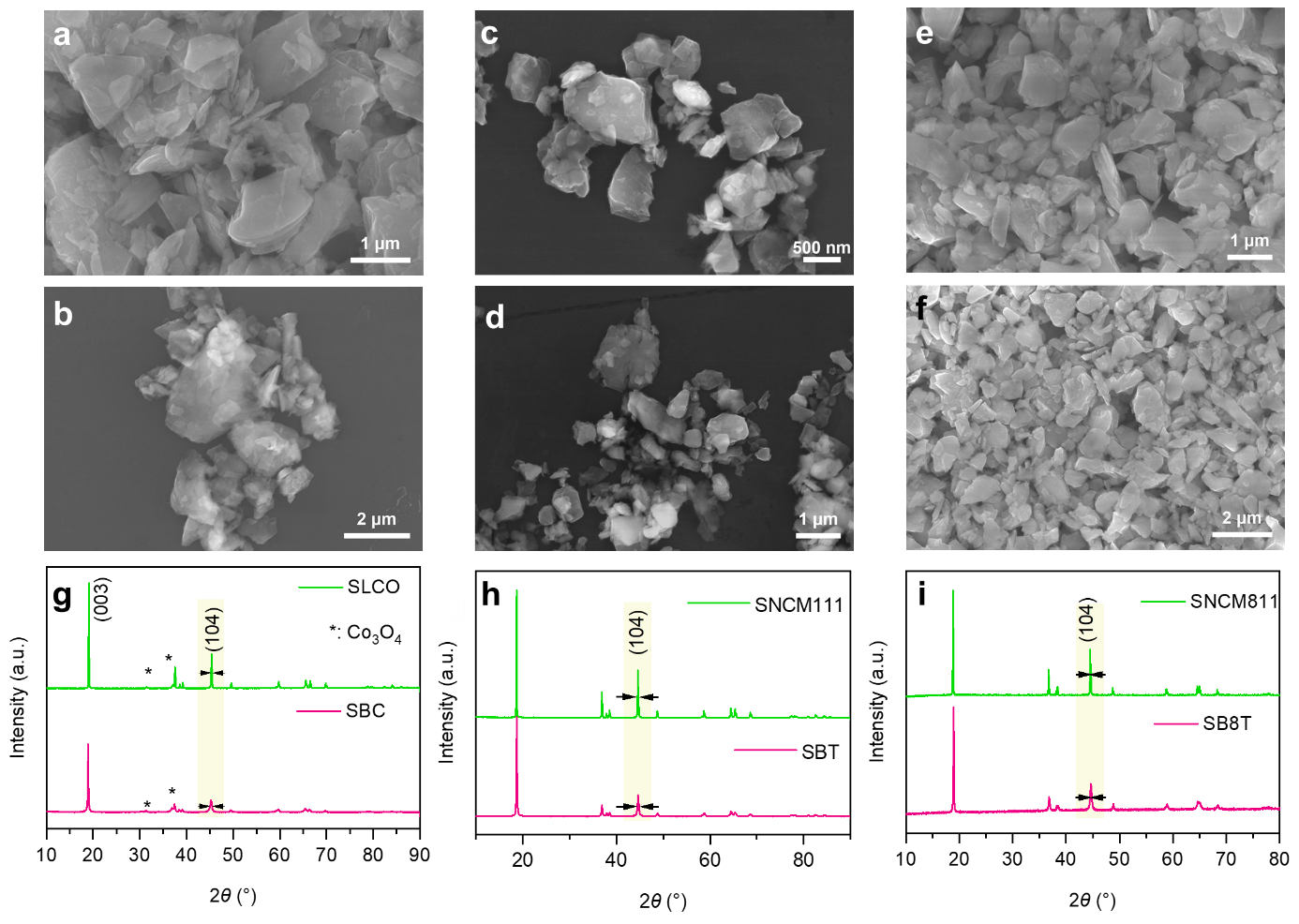
Figure S4.** SEM images for **a, b)** ball-milled SLCO, **c, d)** ball-milled SNCM111 and **e, f)** ball-milled SNCM811; XRD patterns of precursors before and after ball-milling: **g)** SLCO->SBC, **h)** SNCM111->SBT, and **i)** SNCM811->SB8T.


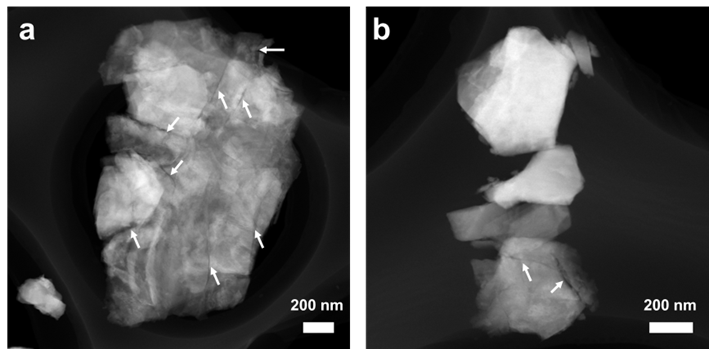


**Figure S5.** **a, b)** HAADF-STEM images for SBT.

**
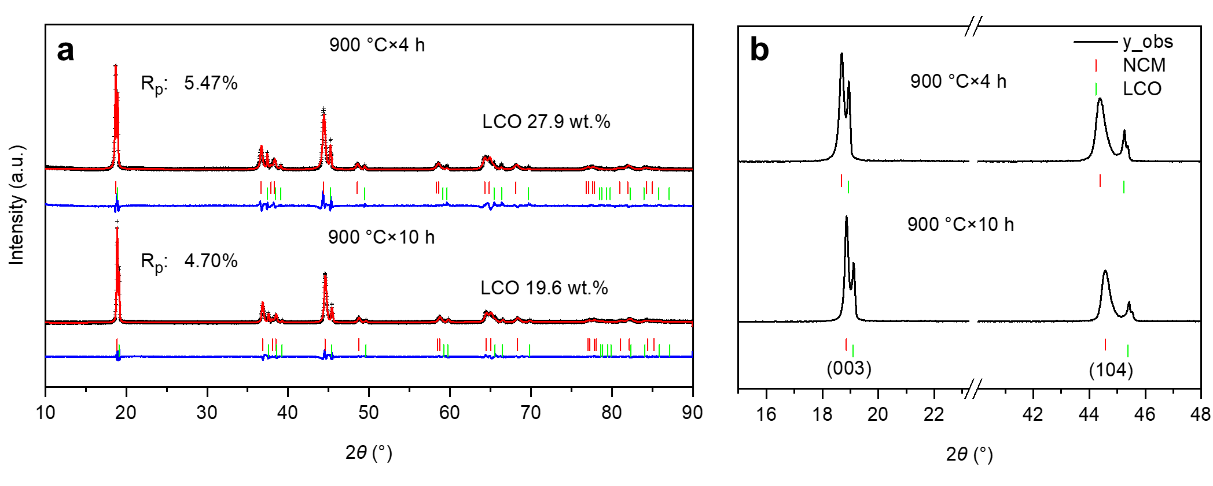
**

**Figure S6.** **a, b)** Rietveld refinement results for NCM111-LCO-MS after calcination for 4 and 10 hours, with selected (003) and (104) peaks enlarged.


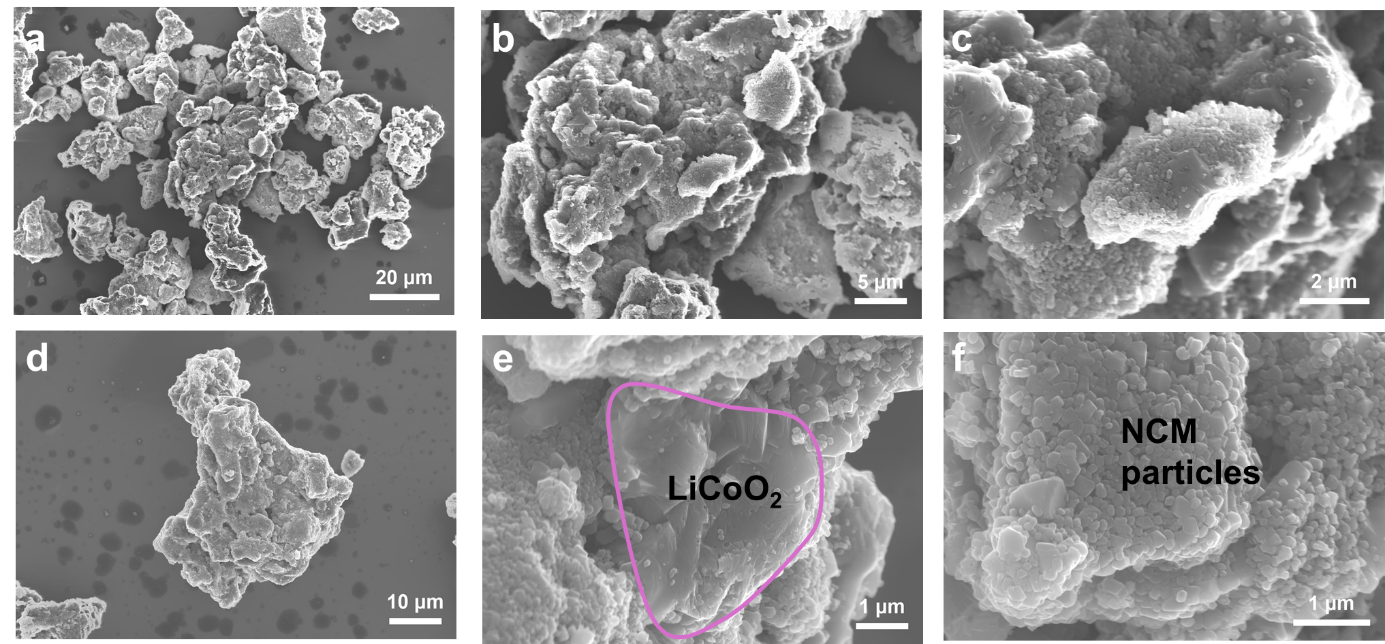
**Figure S7. a-f)** SEM images of NCM111-LCO-MS particles showing a morphology of irregularly shaped particles.

**
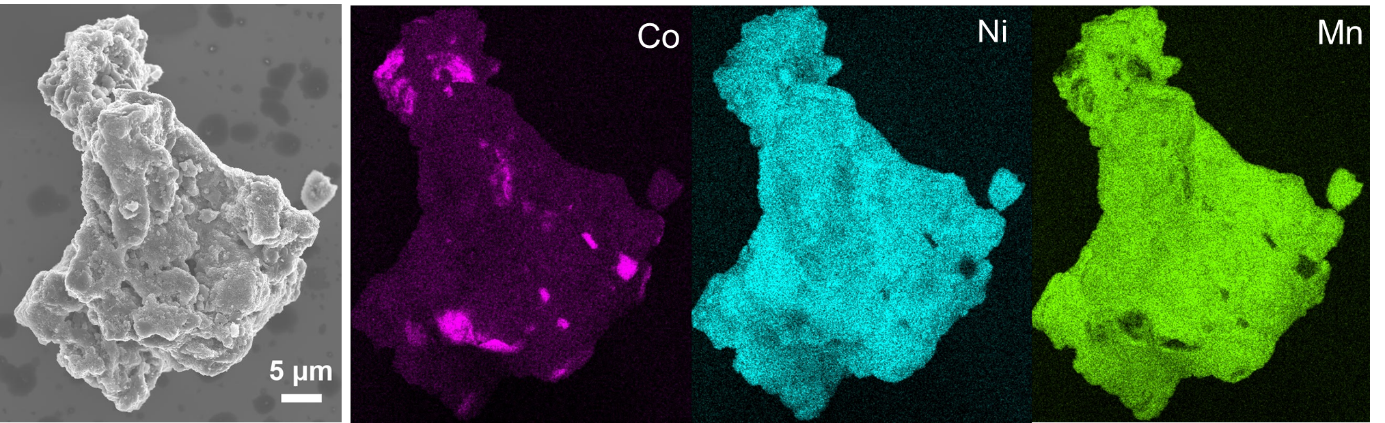
Figure S8.** EDX mapping results for Co, Ni and Mn distribution within NCM111-LCO-MS.

**
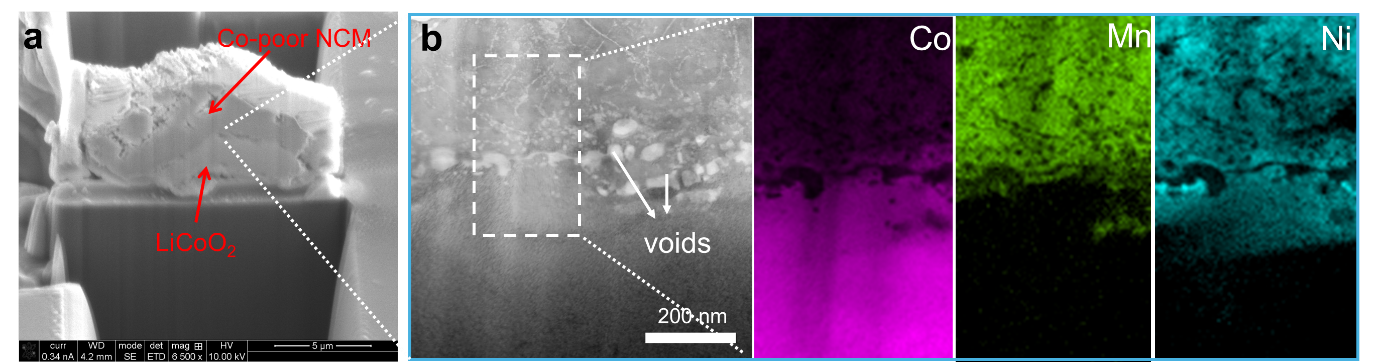
Figure S9.** **a)** FIB-SEM milled cross-sectional imaging of NCM111-LCO-MS samples; **b)** EELS mapping performed under STEM mode for NCM111-LCO-MS.

**
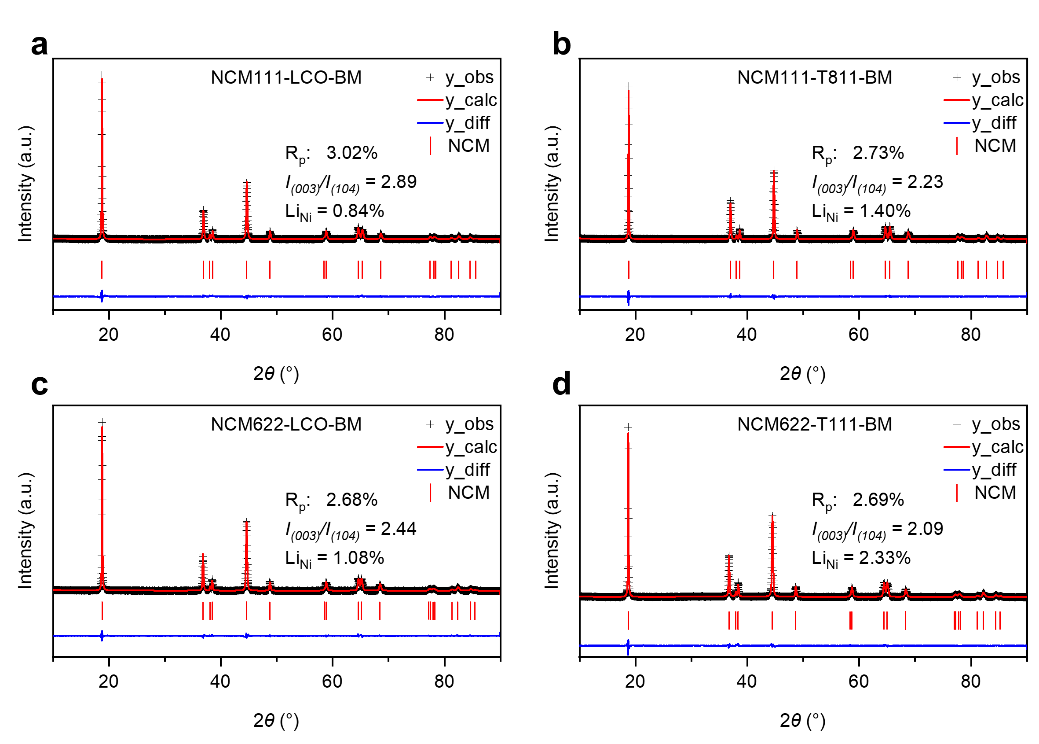
**

**Figure S10.** Rietveld refinement for the XRD results of **a)** NCM111-LCO-BM, **b)** NCM111-T811-BM, **c)** NCM622-LCO-BM and **d)** NCM622-T111-BM.


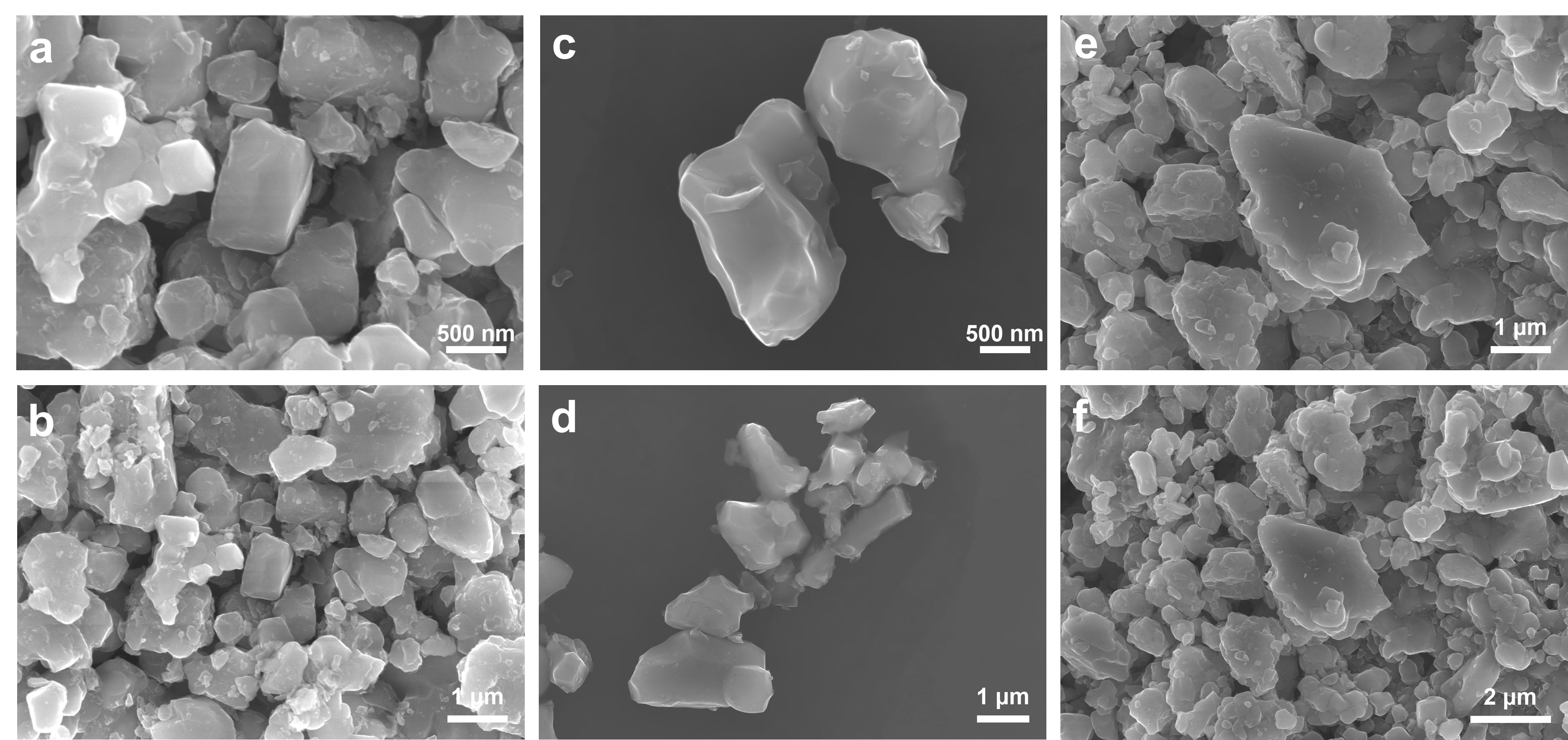


**Figure S11.** SEM images of the upcycled cathodes from LCO precursors: **a, b)** NCM111-LCO-BM; **c, d)** NCM523-LCO-BM; **e, f)** NCM622-LCO-BM.


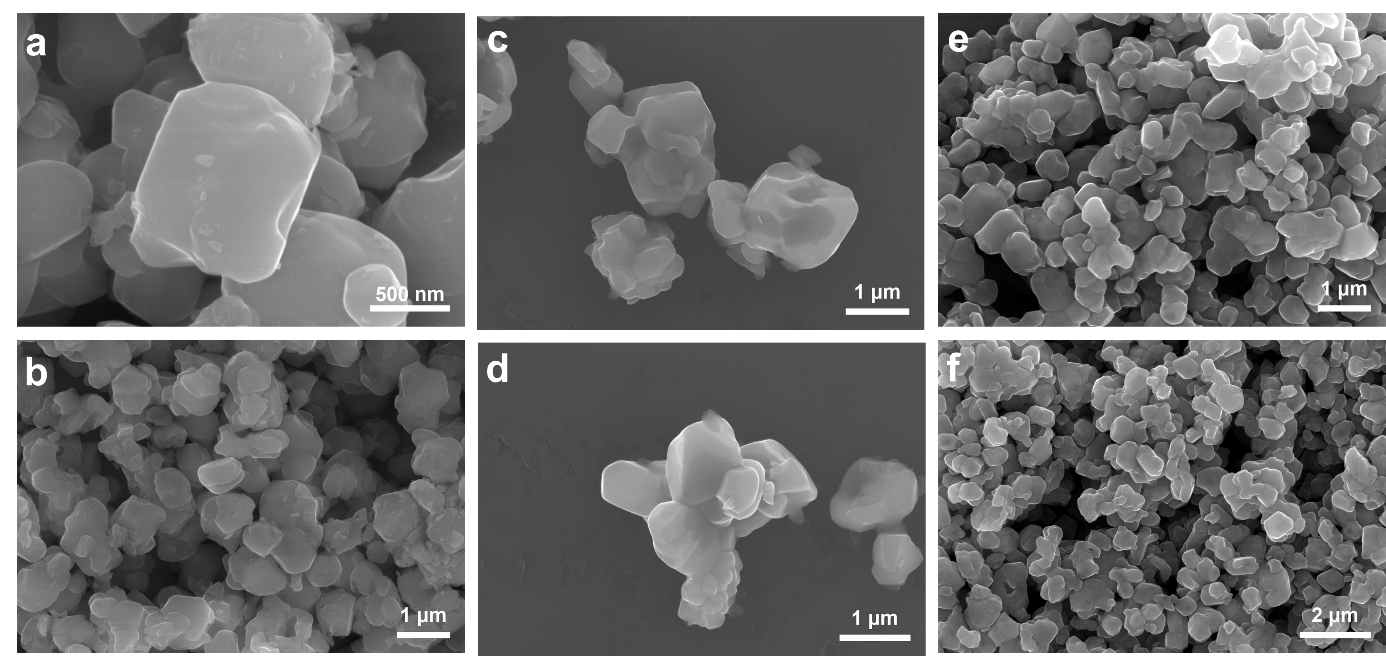


**Figure S12.** SEM images of the upcycled cathodes from NCM precursors: **a, b)** NCM111-T811-BM; **c, d)** NCM523-T111-BM; **e, f)** NCM622-T111-BM.


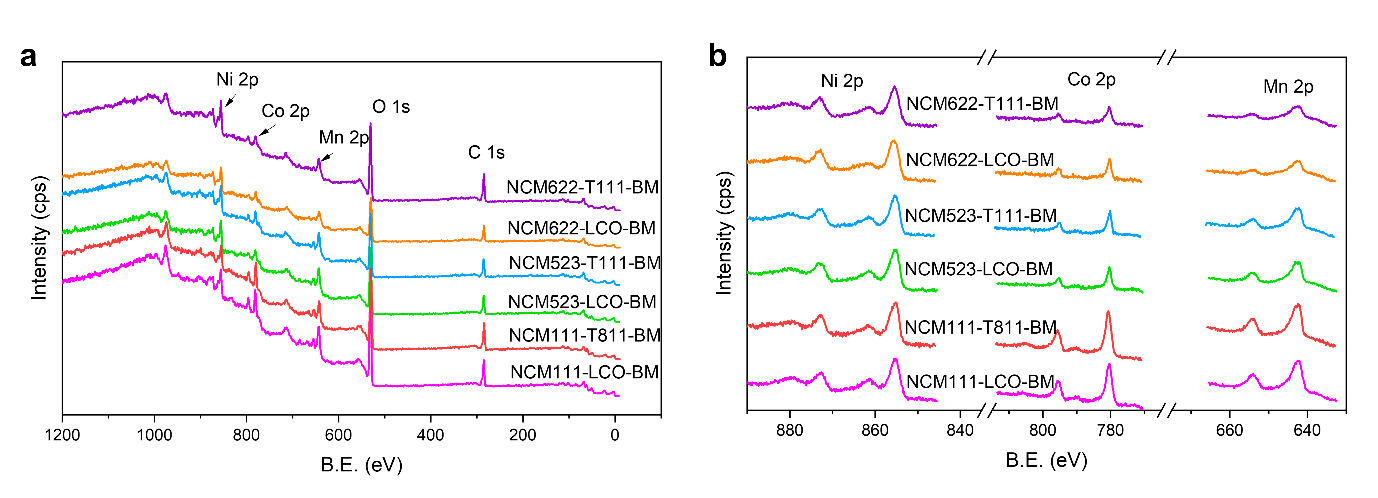


**Figure S13.** **a)** XPS full survey and **b)** high-resolution Ni, Co, and Mn 2p XPS spectra of the upcycled samples.


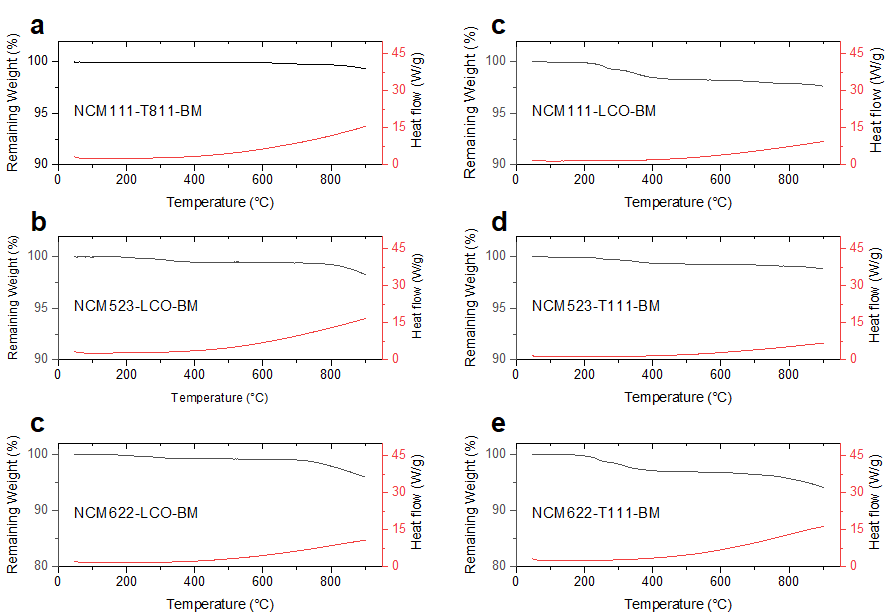


**Figure S14.** Thermal stability of the upcycled cathodes.

**
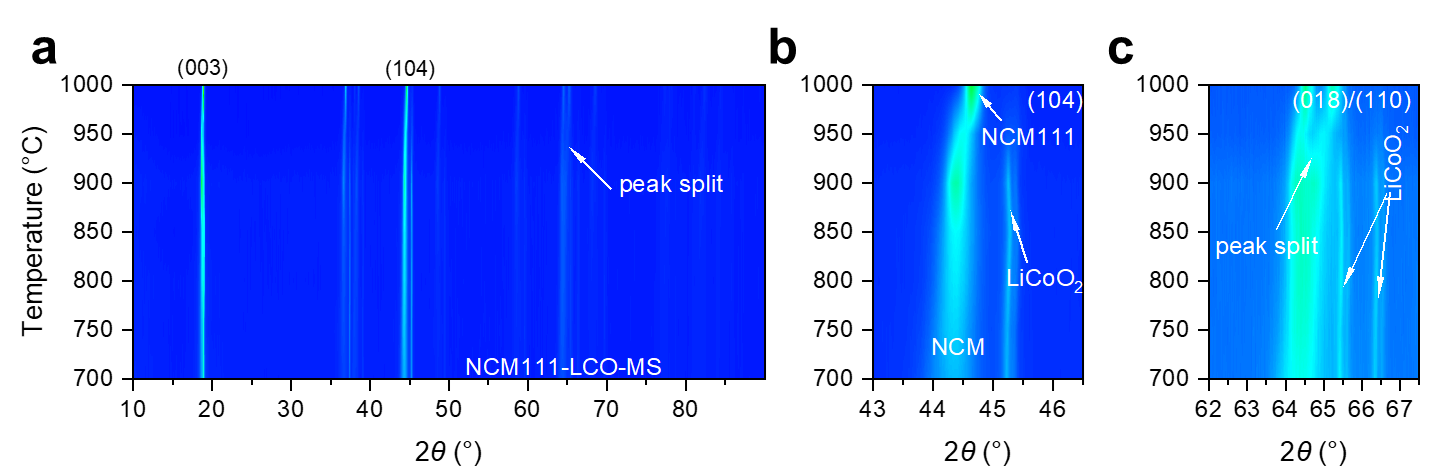
Figure S15.** Ex-situ XRD patterns collected for **a-c)** NCM111-LCO-MS products after calcination under 700-1000 °C, in an interval of 50 °C, for 4 hours each.


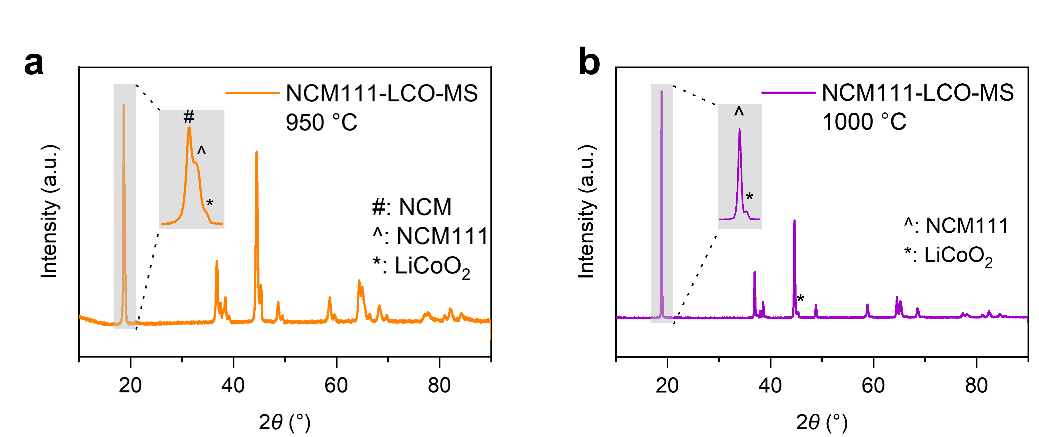


**Figure S16.** XRD patterns of NCM111-LCO-MS processed under **a)** 950 ℃ and **b)** 1000 ℃ for 4 hours.

**
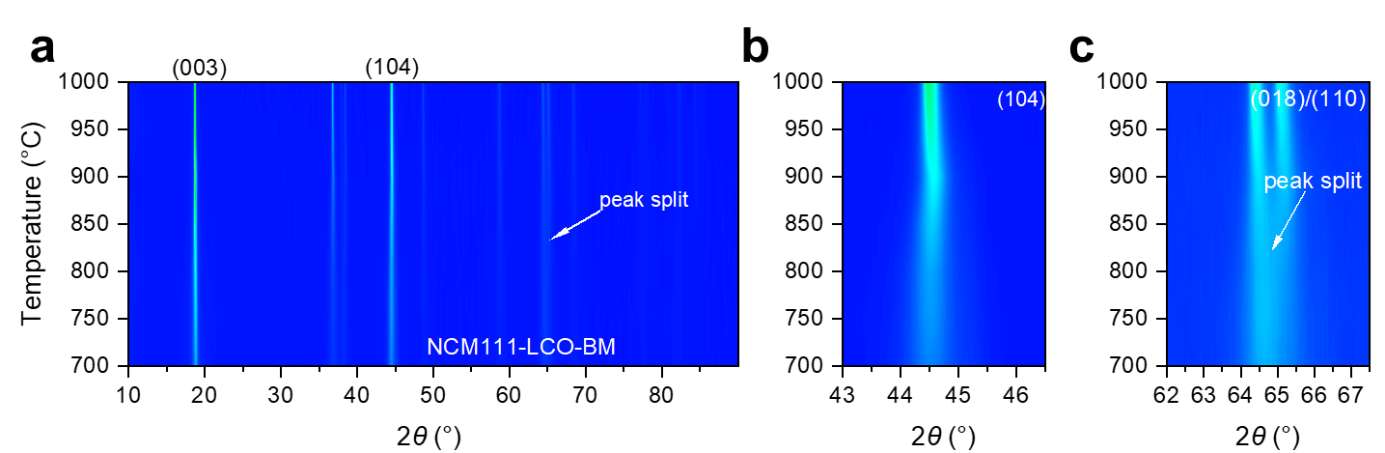
**

**Figure S17.** Ex-situ XRD patterns collected for **a-c)** NCM111-LCO-BM products after calcination under 700-1000 °C, in an interval of 50 °C, for 4 hours each.


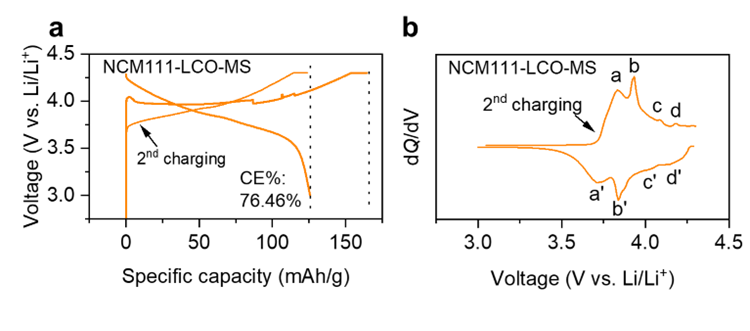


**Figure S18. a)** Voltage profiles and **b)** corresponding differential d*Q*/d*V* plots for NCM111-LCO-MS.

**
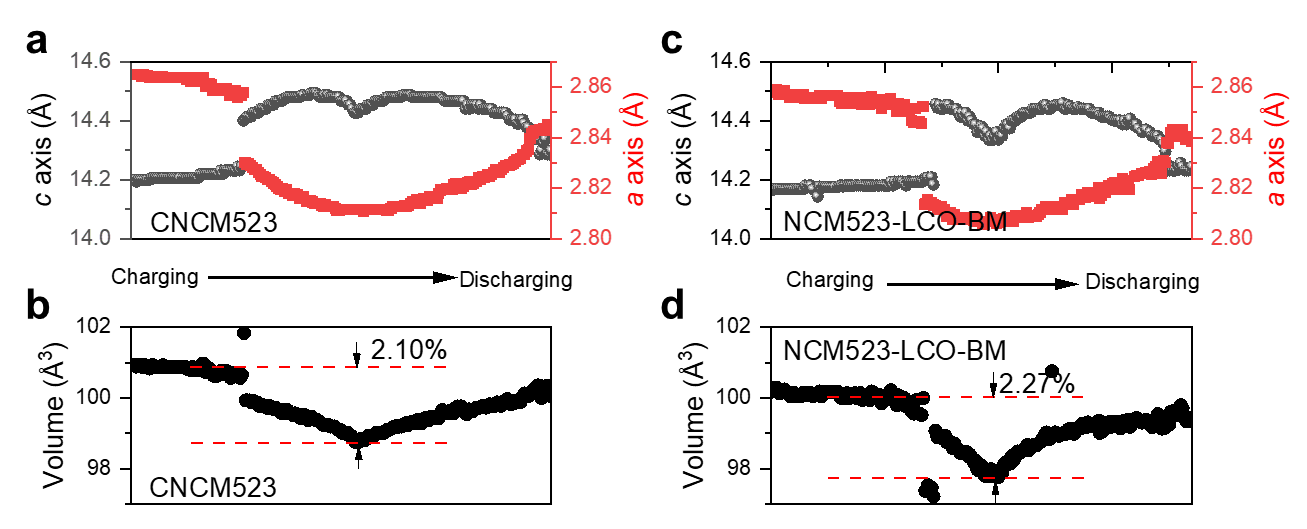
**

**Figure S19.** Calculated lattice parameters (*a* and *c*) change, and lattice volume evolution with charging and discharging process for **a, b)** CNCM523 and **c, d)** NCM523-LCO-BM cathodes.


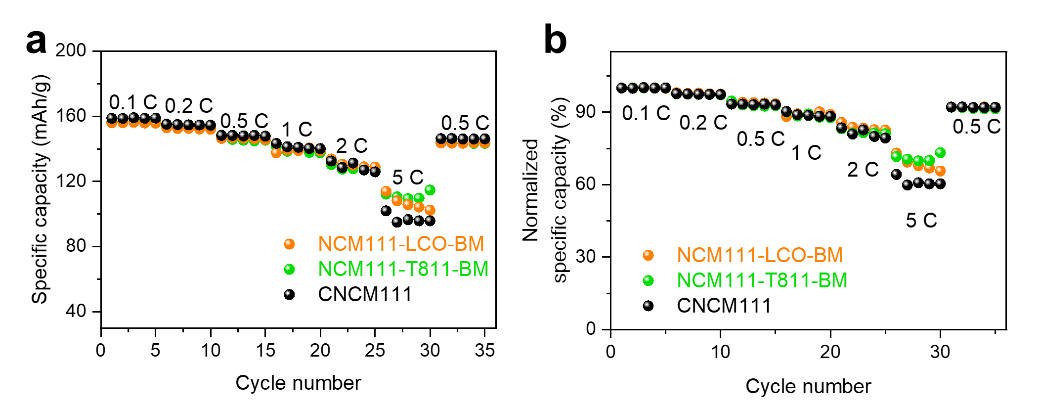


**Figure S20. a)** Rate performances of NCM111-LCO-BM, NCM111-T811-BM vs. CNCM111, and **b)** corresponding normalized plot.

**
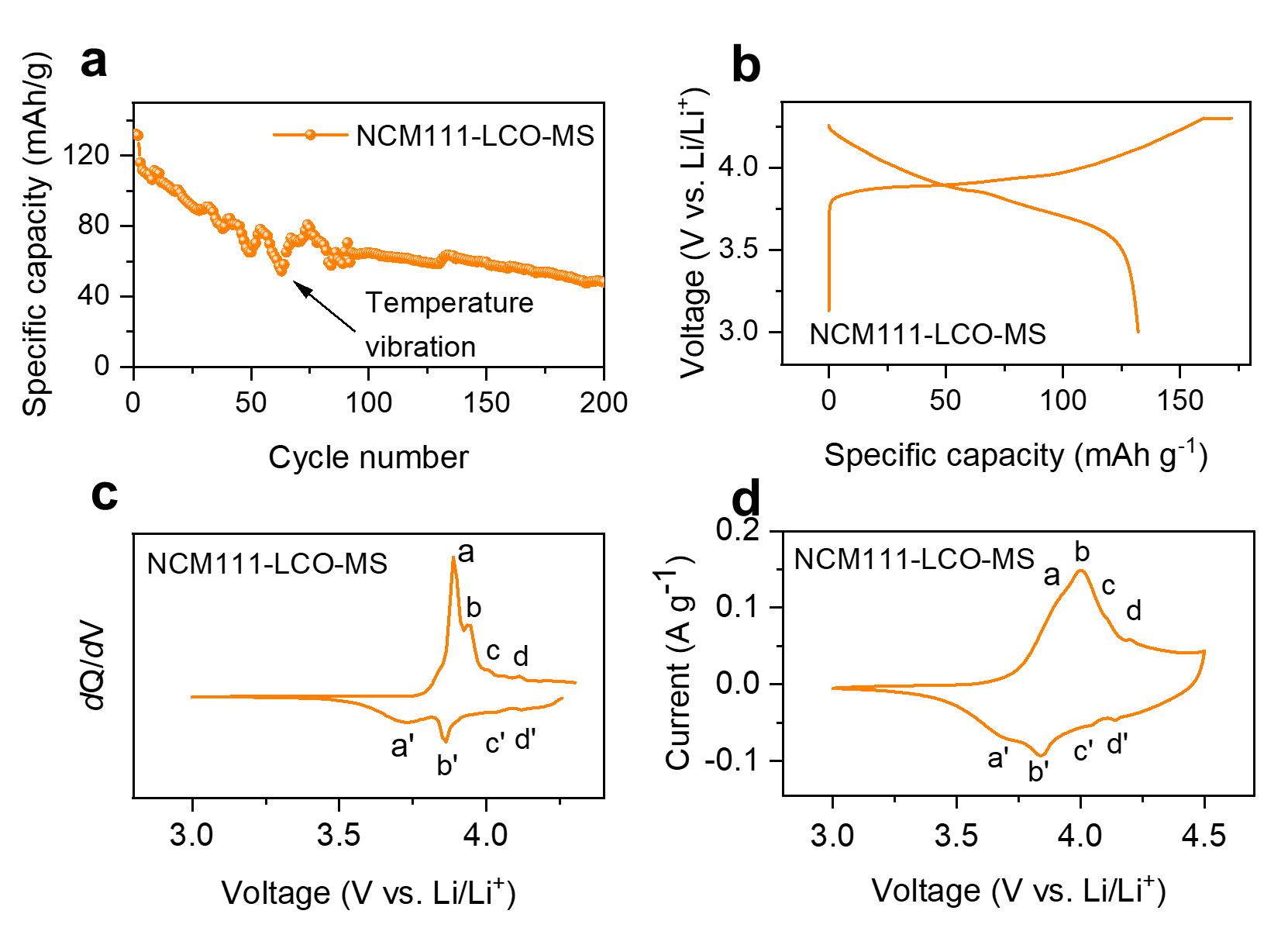
**

**Figure S21. a)** Cycling performance of NCM111-LCO-MS at 0.5 C (100 mA/g); **b)** 1^st^ charging and discharging curves for NCM111-LCO-MS and **c)** corresponding *d*Q/*d*V plots; d) CV curve of NCM111-LCO-MS recorded at 0.1 mV/s.

**
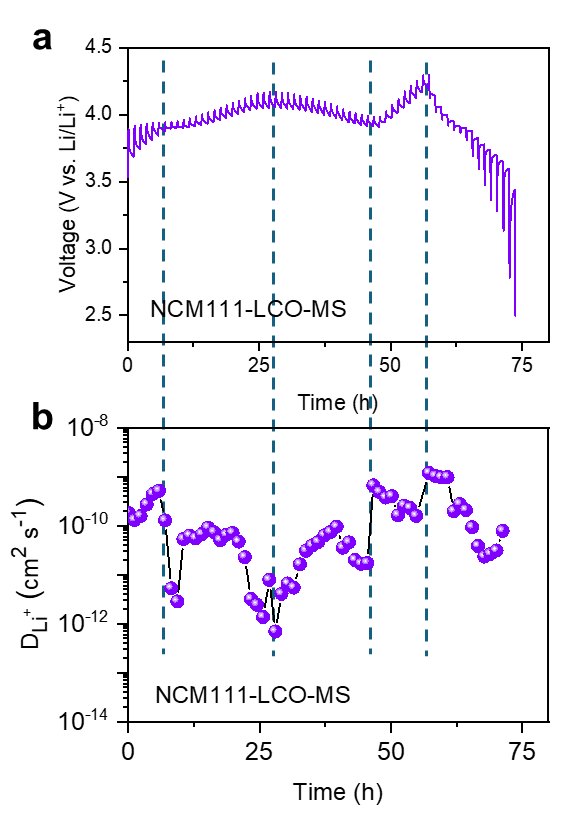
**

**Figure S22.** **a)** GITT curve and **b)** the Li^+^ diffusion coefficient ($D_{{Li}^{+}}$) for NCM111-LCO-MS.


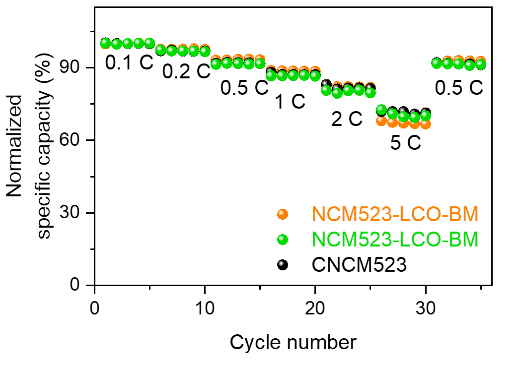


**Figure S23.** Normalized rate performances of NCM523-LCO-BM, NCM523-T111-BM vs. CNCM523.


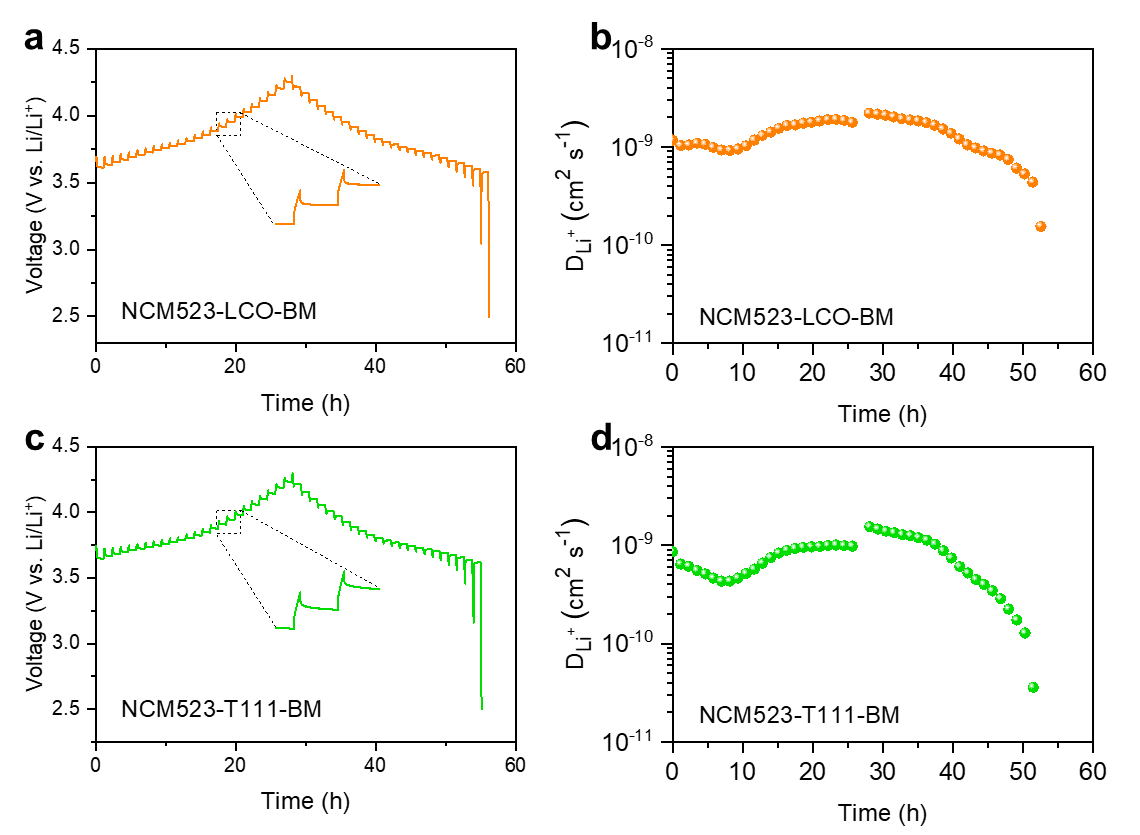


**Figure S24.** **a)** GITT curve and **b)** the Li^+^ diffusion coefficient ($D_{{Li}^{+}}$) for NCM523-LCO-BM; **c)** GITT curve and **d)** the Li^+^ diffusion coefficient ($D_{{Li}^{+}}$) for NCM523-T111-BM.


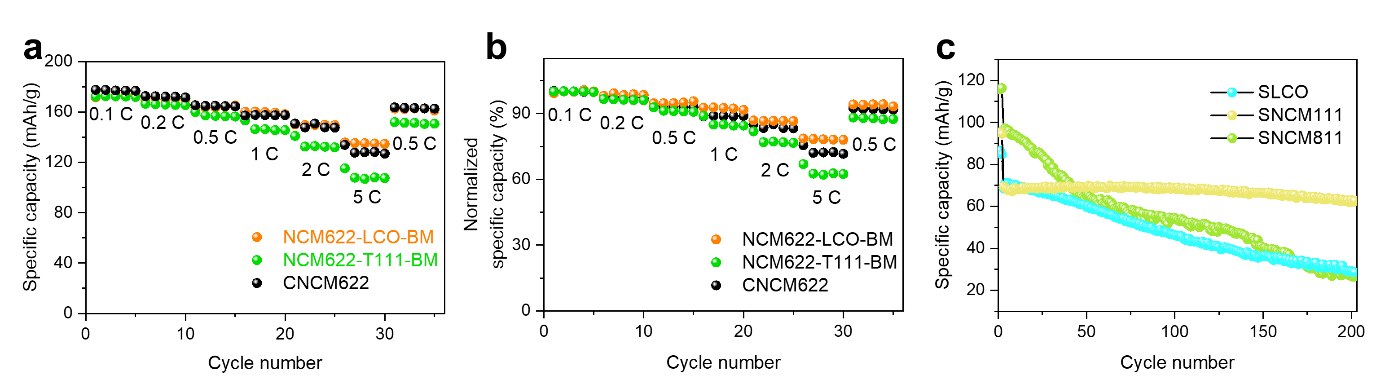


**Figure S25. a)** Rate performances and **b)** normalized rate performance of NCM622-LCO-BM, NCM622-T811-BM vs. CNCM622; **c)** Cycling performance of degraded precursors, SLCO, SNCM111 and SNCM811.

**
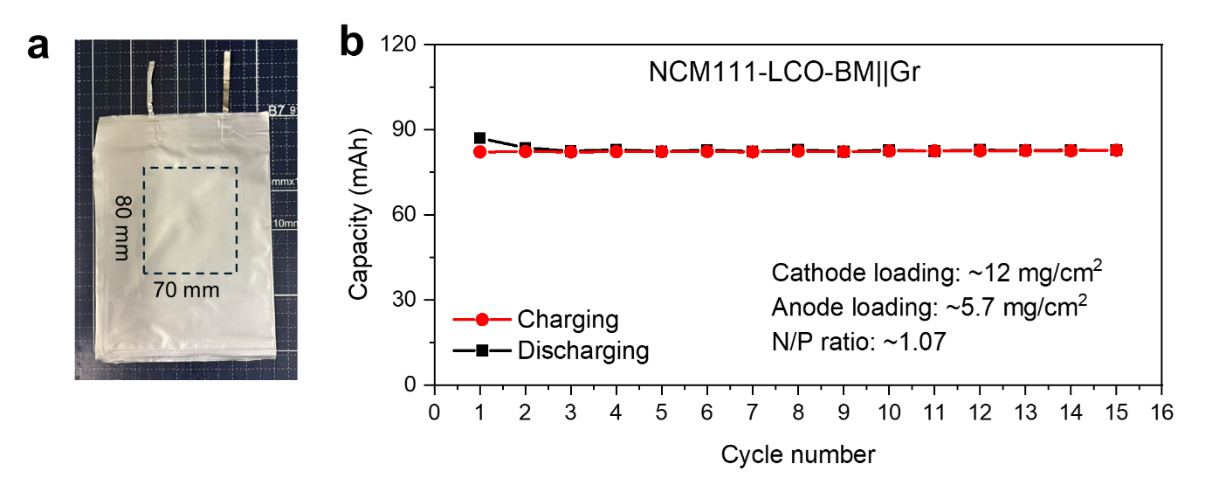
**

**Figure S26.** **a)** Cycling performance for NCM111-LCO-BM||Gr pouch cell; **b)** The assembled pouch cell with electrode dimension in 70 × 80 mm.


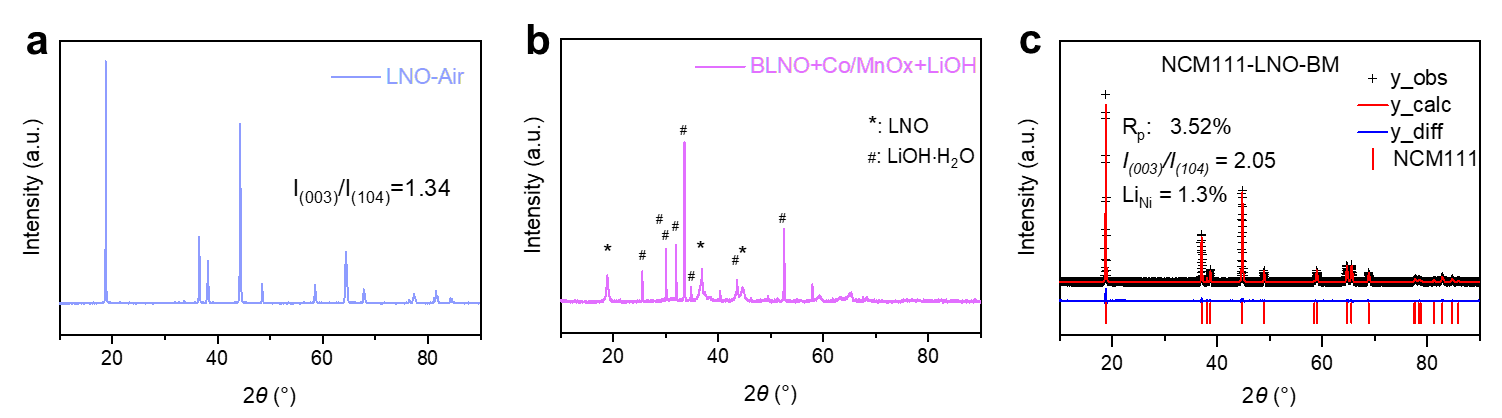


**Figure S27.** Upcycling from LiNiO_2_ down to NCM111-LNO-BM: XRD patterns for **a)** LNO-Air, and NCM111-LNO-BM **b)** before and **c)** after final solid-state sintering.

# Supplemental Tables

**Table S1.** XRD refinement results for NCM111-LCO-MS (900 ℃-4h).

| **NCM111-LCO-MS (900 ℃-4h)** | | | | | | | |
| --- | --- | --- | --- | --- | --- | --- | --- |
| **Phase LiCoO_2_:**  **Atomic Occupancies** | **Atom** | **x** | **y** | | **z** | **Occ.** | **Biso.** |
|  | **Li_3a_** | 0 | 0 | | 0 | 0.976 | 0.0863 |
|  | **Ni_3a_** | 0 | 0 | | 0 | 0.024 | 0.0863 |
|  | **Co_3b_** | 0 | 0 | | 0.5 | 0.929 | 0.0906 |
|  | **Ni_3b_** | 0 | 0 | | 0.5 | 0.071 | 0.0906 |
|  | **O_6c_** | 0 | 0 | | 0.248954 | 1 | 0.0753 |
| **Lattice Parameters** | **a/Å** | **c/Å** | **V/Å^3^** | | **c/a** | **Phase fraction** | |
|  | 2.817476 | 14.0677 | 96.711 | | 4.99 | 27.9 wt.% | |
| **Phase NCM:**  **Atomic Occupancies** | **Atom** | **x** | **y** | | **z** | **Occ.** | **Biso.** |
|  | **Li_3a_** | 0 | 0 | | 0 | 0.931 | 0.005 |
|  | **Ni_3a_** | 0 | 0 | | 0 | 0.069 | 0.005 |
|  | **Li_3b_** | 0 | 0 | | 0.5 | 0.069 | 0.0039 |
|  | **Ni_3b_** | 0 | 0 | | 0.5 | 0.381 | 0.0039 |
|  | **Mn_3b_** | 0 | 0 | | 0.5 | 0.45 | 0.0039 |
|  | **Co_3b_** | 0 | 0 | | 0.5 | 0.1 | 0.0039 |
|  | **O_6c_** | 0 | 0 | | 0.241915 | 1 | 0.002 |
| **Lattice Parameters** | **a/Å** | **c/Å** | **V/Å^3^** | | **c/a** | **Phase fraction** | |
|  | 2.872378 | 14.26153 | 101.901 | | 4.97 | 72.1 wt.% | |
| **Agreement Factors** | | | | | | | |
| **R_p_ = 5.47%** | | | | **R_wp_ = 7.74%** | | | |

**Table S2.** XRD refinement results for NCM111-LCO-MS (900 ℃-10h).

| **NCM111-LCO-MS (900 ℃-10h)** | | | | | | | |
| --- | --- | --- | --- | --- | --- | --- | --- |
| **Phase LiCoO_2_:**  **Atomic Occupancies** | **Atom** | **x** | **y** | | **z** | **Occ.** | **Biso.** |
|  | **Li_3a_** | 0 | 0 | | 0 | 0.957 | 0.1913 |
|  | **Ni_3a_** | 0 | 0 | | 0 | 0.043 | 0.1913 |
|  | **Co_3b_** | 0 | 0 | | 0.5 | 0.962 | 0.0307 |
|  | **Ni_3b_** | 0 | 0 | | 0.5 | 0.038 | 0.0307 |
|  | **O_6c_** | 0 | 0 | | 0.233532 | 1 | 0.0252 |
| **Lattice Parameters** | **a/Å** | **c/Å** | **V/Å^3^** | | **c/a** | **Phase fraction** | |
|  | 2.817082 | 14.06507 | 96.666 | | 4.99 | 19.6 wt.% | |
| **Phase NCM:**  **Atomic Occupancies** | **Atom** | **x** | **y** | | **z** | **Occ.** | **Biso.** |
|  | **Li_3a_** | 0 | 0 | | 0 | 0.939 | 0.0038 |
|  | **Ni_3a_** | 0 | 0 | | 0 | 0.061 | 0.0038 |
|  | **Li_3b_** | 0 | 0 | | 0.5 | 0.061 | 0.0074 |
|  | **Ni_3b_** | 0 | 0 | | 0.5 | 0.369 | 0.0074 |
|  | **Mn_3b_** | 0 | 0 | | 0.5 | 0.43 | 0.0074 |
|  | **Co_3b_** | 0 | 0 | | 0.5 | 0.14 | 0.0074 |
|  | **O_6c_** | 0 | 0 | | 0.240865 | 1 | 0.0019 |
| **Lattice Parameters** | **a/Å** | **c/Å** | **V/Å^3^** | | **c/a** | **Phase fraction** | |
|  | 2.872795 | 14.24561 | 101.817 | | 4.96 | 80.4 wt.% | |
| **Agreement Factors** | | | | | | | |
| **R_p_ = 4.70%** | | | | **R_wp_ = 6.91%** | | | |

**Table S3.** XRD refinement results for NCM111-LCO-BM.

| **NCM111-LCO-BM (Space Group:**$R\bar{3}m$**)** | | | | | | | | | | |
| --- | --- | --- | --- | --- | --- | --- | --- | --- | --- | --- |
| **Atomic Occupancies** | **Atom** | **x** | | **y** | | **z** | | **Occ.** | | **Biso.** |
|  | **Li_3a_** | 0 | | 0 | | 0 | | 0.9916 | | 0.0044 |
|  | **Ni_3a_** | 0 | | 0 | | 0.5 | | 0.0084 | | 0.0044 |
|  | **Li_3b_** | 0 | | 0 | | 0 | | 0.0084 | | 0.0138 |
|  | **Ni_3b_** | 0 | | 0 | | 0.5 | | 0.325 | | 0.0138 |
|  | **Mn_3b_** | 0 | | 0 | | 0.5 | | 0.333 | | 0.0138 |
|  | **Co_3b_** | 0 | | 0 | | 0.5 | | 0.333 | | 0.0138 |
|  | **O_6c_** | 0 | | 0 | | 0.24085 | | 1 | | 0.0255 |
| **Lattice Parameters** | **a/Å** | | **c/Å** | | | | **V/Å^3^** | | **c/a** | |
|  | 2.85714 | | 14.21089 | | | | 100.465 | | 4.97 | |
| **Agreement Factors** | | | | | | | | | | |
| **R_p_ = 3.02%** | | | | | **R_wp_ = 4.66%** | | | | | |

**Table S4.** XRD refinement results for NCM111-T811-BM.

| **NCM111-T811-BM (Space Group:**$R\bar{3}m$**)** | | | | | | | | | | |
| --- | --- | --- | --- | --- | --- | --- | --- | --- | --- | --- |
| **Atomic Occupancies** | **Atom** | **x** | | **y** | | **z** | | **Occ.** | | **Biso.** |
|  | **Li_3a_** | 0 | | 0 | | 0 | | 0.986 | | 0.0207 |
|  | **Ni_3a_** | 0 | | 0 | | 0.5 | | 0.014 | | 0.0207 |
|  | **Li_3b_** | 0 | | 0 | | 0 | | 0.014 | | 0.0149 |
|  | **Ni_3b_** | 0 | | 0 | | 0.5 | | 0.3194 | | 0.0149 |
|  | **Mn_3b_** | 0 | | 0 | | 0.5 | | 0.333 | | 0.0149 |
|  | **Co_3b_** | 0 | | 0 | | 0.5 | | 0.333 | | 0.0149 |
|  | **O_6c_** | 0 | | 0 | | 0.24102 | | 1 | | 0.0182 |
| **Lattice Parameters** | **a/Å** | | **c/Å** | | | | **V/Å^3^** | | **c/a** | |
|  | 2.848134 | | 14.18493 | | | | 99.65 | | 4.98 | |
| **Agreement Factors** | | | | | | | | | | |
| **R_p_ = 2.73%** | | | | | **R_wp_ = 4.29%** | | | | | |

**Table S5.** XRD refinement results for NCM523-LCO-BM.

| **NCM523-LCO-BM (Space Group:**$R\bar{3}m$**)** | | | | | | | | | | |
| --- | --- | --- | --- | --- | --- | --- | --- | --- | --- | --- |
| **Atomic Occupancies** | **Atom** | **x** | | **y** | | **z** | | **Occ.** | | **Biso.** |
|  | **Li_3a_** | 0 | | 0 | | 0 | | 0.983 | | 0.0067 |
|  | **Ni_3a_** | 0 | | 0 | | 0.5 | | 0.017 | | 0.0067 |
|  | **Li_3b_** | 0 | | 0 | | 0 | | 0.017 | | 0.0173 |
|  | **Ni_3b_** | 0 | | 0 | | 0.5 | | 0.483 | | 0.0173 |
|  | **Mn_3b_** | 0 | | 0 | | 0.5 | | 0.3 | | 0.0173 |
|  | **Co_3b_** | 0 | | 0 | | 0.5 | | 0.2 | | 0.0173 |
|  | **O_6c_** | 0 | | 0 | | 0.2407 | | 1 | | 0.0278 |
| **Lattice Parameters** | **a/Å** | | **c/Å** | | | | **V/Å^3^** | | **c/a** | |
|  | 2.865625 | | 14.21478 | | | | 101.09 | | 4.96 | |
| **Agreement Factors** | | | | | | | | | | |
| **R_p_ = 3.27%** | | | | | **R_wp_ = 4.66%** | | | | | |

**Table S6.** XRD refinement results for NCM523-T111-BM.

| **NCM523-T111-BM (Space Group:**$R\bar{3}m$**)** | | | | | | | | | | |
| --- | --- | --- | --- | --- | --- | --- | --- | --- | --- | --- |
| **Atomic Occupancies** | **Atom** | **x** | | **y** | | **z** | | **Occ.** | | **Biso.** |
|  | **Li_3a_** | 0 | | 0 | | 0 | | 0.966 | | 0.013 |
|  | **Ni_3a_** | 0 | | 0 | | 0.5 | | 0.034 | | 0.0131 |
|  | **Li_3b_** | 0 | | 0 | | 0 | | 0.034 | | 0.0094 |
|  | **Ni_3b_** | 0 | | 0 | | 0.5 | | 0.466 | | 0.0094 |
|  | **Mn_3b_** | 0 | | 0 | | 0.5 | | 0.3 | | 0.0094 |
|  | **Co_3b_** | 0 | | 0 | | 0.5 | | 0.2 | | 0.0094 |
|  | **O_6c_** | 0 | | 0 | | 0.24304 | | 1 | | 0.0158 |
| **Lattice Parameters** | **a/Å** | | **c/Å** | | | | **V/Å^3^** | | **c/a** | |
|  | 2.86372 | | 14.21775 | | | | 100.977 | | 4.96 | |
| **Agreement Factors** | | | | | | | | | | |
| **R_p_ = 2.91%** | | | | | **R_wp_ = 3.72%** | | | | | |

**Table S7.** XRD refinement results for NCM622-LCO-BM.

| **NCM622-LCO-BM (Space Group:**$R\bar{3}m$**)** | | | | | | | | | | |
| --- | --- | --- | --- | --- | --- | --- | --- | --- | --- | --- |
| **Atomic Occupancies** | **Atom** | **x** | | **y** | | **z** | | **Occ.** | | **Biso.** |
|  | **Li_3a_** | 0 | | 0 | | 0 | | 0.9892 | | 0.0064 |
|  | **Ni_3a_** | 0 | | 0 | | 0.5 | | 0.0108 | | 0.0064 |
|  | **Li_3b_** | 0 | | 0 | | 0 | | 0.0108 | | 0.01898 |
|  | **Ni_3b_** | 0 | | 0 | | 0.5 | | 0.3371 | | 0.0252 |
|  | **Mn_3b_** | 0 | | 0 | | 0.5 | | 0.2 | | 0.01898 |
|  | **Co_3b_** | 0 | | 0 | | 0.5 | | 0.2 | | 0.01898 |
|  | **O_6c_** | 0 | | 0 | | 0.24033 | | 1 | | 0.0236 |
| **Lattice Parameters** | **a/Å** | | **c/Å** | | | | **V/Å^3^** | | **c/a** | |
|  | 2.862549 | | 14.18555 | | | | 100.666 | | 4.96 | |
| **Agreement Factors** | | | | | | | | | | |
| **R_p_ = 2.68%** | | | | | **R_wp_ = 4.19%** | | | | | |

**Table S8.** XRD refinement results for NCM622-T111-BM.

| **NCM622-T111-BM (Space Group:**$R\bar{3}m$**)** | | | | | | | | | | |
| --- | --- | --- | --- | --- | --- | --- | --- | --- | --- | --- |
| **Atomic Occupancies** | **Atom** | **x** | | **y** | | **z** | | **Occ.** | | **Biso.** |
|  | **Li_3a_** | 0 | | 0 | | 0 | | 0.977 | | 0.0071 |
|  | **Ni_3a_** | 0 | | 0 | | 0.5 | | 0.023 | | 0.0071 |
|  | **Li_3b_** | 0 | | 0 | | 0 | | 0.023 | | 0.01517 |
|  | **Ni_3b_** | 0 | | 0 | | 0.5 | | 0.576692 | | 0.01517 |
|  | **Mn_3b_** | 0 | | 0 | | 0.5 | | 0.2 | | 0.01517 |
|  | **Co_3b_** | 0 | | 0 | | 0.5 | | 0.2 | | 0.01517 |
|  | **O_6c_** | 0 | | 0 | | 0.75834 | | 1 | | 0.0248 |
| **Lattice Parameters** | **a/Å** | | **c/Å** | | | | **V/Å^3^** | | **c/a** | |
|  | 2.866752 | | 14.21385 | | | | 101.163 | | 4.96 | |
| **Agreement Factors** | | | | | | | | | | |
| **R_p_ = 2.69%** | | | | | **R_wp_ = 3.56%** | | | | | |

**Table S9.** High-resolution Ni 2p XPS spectra fitting results.

| Sample | Ni 2p_3/2_ location (eV) | Valence | Location (eV) | FWHM | Area | Ni^3+^/Ni^2+^ ratio |
| --- | --- | --- | --- | --- | --- | --- |
| **NCM111-LCO-BM** | 855.39 | Ni^2+^ | 855.10 | 1.91 | 7493.89 | 1.45 |
|  |  | Ni^3+^ | 856.10 | 3.71 | 10867.52 |  |
| **NCM111-T811-BM** | 855.48 | Ni^2+^ | 855.10 | 1.76 | 5615.40 | 1.28 |
|  |  | Ni^3+^ | 856.0 | 2.76 | 7167.22 |  |
| **NCM523-LCO-BM** | 855.29 | Ni^2+^ | 855.10 | 1.77 | 6750.78 | 1.53 |
|  |  | Ni^3+^ | 856.10 | 3.06 | 10299.69 |  |
| **NCM523-T111-BM** | 855.46 | Ni^2+^ | 855.10 | 1.85 | 7514.76 | 1.09 |
|  |  | Ni^3+^ | 856.10 | 3.01 | 8169.62 |  |
| **NCM622-LCO-BM** | 855.69 | Ni^2+^ | 855.05 | 1.78 | 4360.57 | 1.80 |
|  |  | Ni^3+^ | 856.10 | 2.58 | 7854.37 |  |
| **NCM622-T111-BM** | 855.50 | Ni^2+^ | 855.10 | 2.03 | 7287.99 | 1.21 |
|  |  | Ni^3+^ | 856.10 | 2.88 | 8850.94 |  |

**Table S10.** Elemental compositions of upcycled cathodes

| **Sample** | Ni (mol.%) | **Co** (mol.%) | **Mn** (mol.%) |
| --- | --- | --- | --- |
| NCM111-LCO-BM | 0.33562 | 0.33495 | 0.32943 |
| NCM111-T811-BM | 0.3425 | 0.3212 | 0.3363 |
| NCM523-LCO-BM | 0.51349 | 0.20824 | 0.27827 |
| NCM523-T111-BM | 0.47309 | 0.19916 | 0.32775 |
| NCM622-LCO-BM | 0.5803 | 0.20749 | 0.21221 |
| NCM622-T111-BM | 0.55491 | 0.20054 | 0.24455 |

**Table S11.** Energy density computation data for NCM111-LCO-BM||Gr pouch cell assembled in this study.

| **Battery type** | **Name** | **Value** | **Mass** | |
| --- | --- | --- | --- | --- |
| NCM111-LCO-BM\|\|Gr | Cathode loading (85 wt.%) | 12 mg/cm^2^ | 790.59 mg | |
|  | Al current collector | 13 μm | 181.44 mg | |
|  | Anode loading (85wt.%) | 5.7 mg/cm^2^ | 375.53 mg | |
|  | Cu current collector | 6 μm | 301.06 mg | |
|  | Electrode dimension | 7 × 8 cm |  | |
|  | Average voltage | 3.7 V | Energy | 305.25 mWh |
|  | Capacity (0.5 C) | 82.5 mAh |  |  |
|  | 185.18 Wh/kg | | | |

**Table S12.** XRD refinement results for NCM111-LNO-BM.

| **NCM111-LNO-BM (Space Group:**$R\bar{3}m$**)** | | | | | | | | | | |
| --- | --- | --- | --- | --- | --- | --- | --- | --- | --- | --- |
| **Atomic Occupancies** | **Atom** | **x** | | **y** | | **z** | | **Occ.** | | **Biso.** |
|  | **Li_3a_** | 0 | | 0 | | 0 | | 0.987 | | 0.011 |
|  | **Ni_3a_** | 0 | | 0 | | 0.5 | | 0.023 | | 0.0071 |
|  | **Li_3b_** | 0 | | 0 | | 0 | | 0.013 | | 0.0112 |
|  | **Ni_3b_** | 0 | | 0 | | 0.5 | | 0.32 | | 0.0115 |
|  | **Mn_3b_** | 0 | | 0 | | 0.5 | | 0.333 | | 0.0115 |
|  | **Co_3b_** | 0 | | 0 | | 0.5 | | 0.333 | | 0.0115 |
|  | **O_6c_** | 0 | | 0 | | 0.24078 | | 1 | | 0.0089 |
| **Lattice Parameters** | **a/Å** | | **c/Å** | | | | **V/Å^3^** | | **c/a** | |
|  | 2.848922 | | 14.21115 | | | | 99.89 | | 4.99 | |
| **Agreement Factors** | | | | | | | | | | |
| **R_p_ = 3.52%** | | | | | **R_wp_ = 4.76%** | | | | | |

**Table S13.** Technoeconomic analysis on the Hydrometallurgy.

|  | **Chemical** | **Amount** | **Unit price** | **Price (US$)** |
| --- | --- | --- | --- | --- |
| **Precursor** | Li_0.6_CoO_2_ | 1 t | 5278.57 US$/t | 5278.57 |
| **Leaching** | H_2_SO_4_ (98%) | 1.64 t | 66.95 US$/t | 109.80 |
|  | H_2_O_2_ | 0.30 t | 615 US$/t | 184.5 |
|  | Electricity: | 775.19 kWh | 0.15 US$/kWh | 116.28 |
|  | Condition: 80 °C, 400 rpm, solid-to-liquid (S/L) ratio: 10%, 98% leaching rate, 90% heating efficiency, 0.2 times excess | | | |
| **Solvent extraction** | NaOH | 0.20 t | 385.34 US$/t | 77.068 |
|  | Na_2_CO_3_ | 0.52 t | 708.12 US$/t | 368.22 |
|  | H_2_SO_4_ | 0.72 t | 66.95 US$/t | 48.20 |
| **Wastewater** |  | 9 t | 50 US$/t | 450 |
| **Final product** | CoSO_4_·7H_2_O | 2.84 t | 4038.28 US$/t | 11468.72 |
|  | Li_2_CO_3_ | 0.18 t | 10756.89 US$/t | 1936.24 |
|  | 98% separation rate for Co, 80% separation rate for Li | | | |

Source: Shanghai Metal Marke (www.metal.com), [www.source.benchmarkminerals.com](http://www.source.benchmarkminerals.com)

${Li}_{0.6}{CoO}_{2}+1.3H_{2}SO_{4}+0.7H_{2}O_{2}\to0.3{Li}_{2}SO_{4}+CoSO_{4}+2H_{2}O+0.7O_{2}$ **Equation S1**

**Table S14.** Technoeconomic analysis on the Hydro-Resynthesis.

|  | **Chemical** | **Amount** | **Unit price** | **Price (US$)** |
| --- | --- | --- | --- | --- |
| **Precursor** | Li_0.6_CoO_2_ | 1 t | 5278.57 US$/t | 5278.57 |
| **Leaching** | H_2_SO_4_ (98%) | 1.64 t | 66.95 US$/t | 109.80 |
|  | H_2_O_2_ | 0.30 t | 615 US$/t | 184.5 |
|  | Electricity: | 775.19 kWh | 0.15 US$/kWh | 116.28 |
|  | Condition: 80 °C, 400 rpm, solid-to-liquid (S/L) ratio: 10%, 98% leaching rate, 90% heating efficiency, 0.2 times excess | | | |
| **Co-precipitation** | NiSO_4_·6H_2_O | 8.13 | 3975 | 32303.01 |
|  | MnSO_4_·H_2_O | 1.74 t | 890.13 | 1550.49 |
|  | NaOH | 2.06 t | 385.34 | 794.24 |
|  | NH_4_OH | 0.15 t | 771.89 US$/t | 115.78 |
|  | N_2_ | 0.5 t |  | 50 |
| **Wastewater** |  | 10.0 t | 50 US$/t | 500 |
| **Resynthesis** | LiOH·H_2_O | 2.27 t | 9765.1 US$/t | 22169.10 |
|  | O_2_ | 4.0 t | 130 US$/t | 720 |
|  | Electricity | 12000 kWh | 0.15 US$/kWh | 1800 |
| **Final product** | NCM622 | 5.00 t | 18285.71 US$/t | 91428.57 |
|  | Li_2_CO_3_ | 0.18 t | 10756.89 US$/t | 1936.24 |
|  | 98% separation rate for Co, 80% separation rate for Li | | | |

**Table S15.** Technoeconomic analysis on the Upcycling (this work).

|  | **Chemical** | **Amount** | **Unit price** | **Price (US$)** |
| --- | --- | --- | --- | --- |
| **Precursor** | Li_0.6_CoO_2_ | 1 t | 5278.57 US$/t | 5278.57 |
| **Ball milling** | Electricity | 160 kWh | 0.15 US$/kWh | 24 |
| **Solid-state sintering** | NiAc_2_·4H_2_O | 7.85 t | 2934.33 US$/t | 23035.68 |
|  | MnAc_2_·4H_2_O | 2.58 t | 839.83 US$/t | 2164.55 |
|  | LiOH·H_2_O | 2.08 t | 9765.1 US$/t | 20311.41 |
|  | O_2_ | 0.8 t | 130 US$/t | 104 |
|  | Electricity | 2400 kWh | 0.15 US$/kWh | 360 |
| **Wastewater** |  | 0 |  | 0 |
| **Final product** | NCM622 | 5.10 t | 18285.71 US$/t | 93257.14 |

It should be noted that only essential consumable chemicals are calculated into economic analysis, to which the price varies greatly, and cost from instrument, labor, transportation, etc., is not considered. The technoeconomic analysis is only intended to be demonstrated for comparison, thus, the values, especially for profit, can vary greatly according to market change.

|  | **Hydrometallurgy** | **Hydro-Resynthesis** | **Upcycling**  **(this work)** |
| --- | --- | --- | --- |
| Electricity (kWh/t) | 777.19 | 12775.19 | 2560 |
| Water (t/t) | 10 | 25.5 | 0 |
| Profit (k US$/t) | 6.77 | 26.9 | 35.02 |
| Complexity | 2.5 | 5 | 2 |
| GHG emission (t/t) | 0.64 | 10.48 | 2.10 |

**Table S16.** Comprehensive comparison between Hydrometallurgy, Hydro-Resynthesis, and Upcycling (this work) approaches.

# References

[1] B. Toby, R. Dreele, GSAS-II: the genesis of a modern open-source all purpose crystallography software package, *J. Appl. Crystallogr.*, **2013**, *46, 544-549.*
